# Distinct immune microenvironment in HER2-low triple-negative breast cancer underlies inferior response to immunotherapy

**DOI:** 10.1038/s41698-026-01562-3

**Published:** 2026-06-17

**Authors:** Hewei Ge, Yuhan Wei, Yalong Qi, Hongnan Mo, Jingtong Zhai, Cheng Zeng, Xiaoying Sun, Fei Ma

**Affiliations:** 1 Department of Medical Oncology, National Cancer Center/National Clinical Research Center for Cancer/Cancer Hospital, Chinese Academy of Medical Sciences and Peking Union Medical College, Beijing, China; 2 Department of Surgery, School of Clinical Medicine, Li Ka Shing Faculty of Medicine, The University of Hong Kong, Hong Kong SAR, China; 3 Department of Oncology, Beijing Friendship Hospital, Capital Medical University, Beijing, China; 4 State Key Laboratory of Molecular Oncology, National Cancer Center/National Clinical Research Center for Cancer/Cancer Hospital, Chinese Academy of Medical Sciences and Peking Union Medical College, Beijing, China; 5 Department of Medical Oncology, Cancer Hospital of HuanXing ChaoYang District, Beijing, China

**Keywords:** Cancer, Immunology, Oncology

## Abstract

Effective predictive biomarkers for immunotherapy in breast cancer remain elusive, limiting precision treatment strategies. Although the biological significance of HER2‑low expression has gained increasing attention, its impact on immunotherapy response remains unknown. Here, we investigated the impact of HER2 expression on response to neoadjuvant immunotherapy in triple-negative breast cancer (TNBC) and explored the underlying biological patterns through comprehensive multi-omics analysis. A multi-center real-world cohort study revealed that HER2-low TNBC patients had poorer responses to immunotherapy than HER2-0 patients, whereas no significant difference was observed in chemotherapy alone; these findings were validated in independent prospective cohorts (ISPY-2 and NeoTRIP). Single‑cell multi‑omics further demonstrated that HER2‑low tumors exhibited reduced expression of major histocompatibility complex and immune checkpoint molecules, along with diminished T‑cell activity, relative to HER2‑0 tumors. Following immunotherapy, HER2‑low tumors showed no significant downregulation of targeted immune checkpoint molecules and less pronounced T‑cell clonal remodeling. Collectively, these findings establish HER2‑low expression as a negative predictor of immunotherapy response, shaped by a less immunogenic tumor microenvironment. These results provide important insights into the distinct immunological features of HER2‑low triple‑negative breast cancer and warrant further mechanistic and clinical investigations.

## Introduction

Triple-negative breast cancer (TNBC) is recognized as the breast cancer subtype with the worst prognosis^1,2^. Owing to its unique molecular profiles, TNBC has traditionally posed complex therapeutic dilemmas; there are limited treatment options for TNBC^2–4^, and chemotherapy remains one of the primary treatment modalities^2^. The emergence of immunotherapy has revolutionized the neoadjuvant treatment landscape for patients with TNBC, increasing pathological complete response (pCR) rates and improving survival outcomes^5–11^. However, approximately 40% of TNBC patients still have residual disease after receiving a combination of neoadjuvant immunotherapy and chemotherapy^5–9^. Existing predictive biomarkers, such as PD-L1, have shown limited predictive value in TNBC patients receiving neoadjuvant ICIs combined with chemotherapy, with unstable performance across different trials^9,11^. Thus, better predictive biomarkers are needed for precise immunotherapy in TNBC patients.

In TNBC, there exists a subgroup of patients with HER2 IHC 1+ results, or IHC 2+ with a negative FISH assay—namely, HER2-low breast cancer^12^. In clinical practice, these tumors are reported as HER2 negative and were often treated the same way as HER2 IHC 0 tumors^2,12^ before HER2-specific antibody-drug conjugates were introduced^13,14^. However, recent studies revealed that HER2-low patients exhibit longer survival times than HER2-0 (also referred to as HER2-0 in this article) patients under the same neoadjuvant treatments, regardless of ER status^15,16^, whereas HER2-low patients have significantly lower neoadjuvant pCR rates than HER2-0 patients in ER-positive patients (Luminal) and similar pCR rates with HER2-0 patients in ER-negative patients (TNBC)^15^. However, among TNBC patients included in a previous study on neoadjuvant pCR, none were receiving immunotherapy^15,16^, and whether differences in neoadjuvant immunotherapy outcomes exist between HER2-low patients and HER2-0 patients remains unclear.

Furthermore, the rapid advancement of high-throughput sequencing and multi-omics analytical methods has spurred growing research into the biological characteristics of HER2-low breast cancer, which has confirmed that HER2-low tumors exhibit distinct molecular features compared to HER2-0 tumors^17–20^. However, current biological investigations remain largely confined to bioinformatics analyzes based on gene expression profiling, lacking in-depth exploration of the tumor microenvironment—particularly from the perspective of immune cell composition, functional states, and cellular interactions. A previous single-cell sequencing-based analysis of pretreatment TNBC samples revealed that HER2-0 patients exhibited signatures associated with increased immune activity and a potentially more active immune microenvironment^20^. However, the conclusions were not robust because of the small sample size (3 HER2-low patients vs. 4 HER2-0 patients) and the lack of external validation datasets. Moreover, the dynamic changes in tumors and the TME after immunotherapy and their differences from those after chemotherapy have not yet been characterized. These factors significantly limit the understanding of the biological explanations of the observed clinical phenomena. Thus, a large-scale, multi-omics analysis is critically needed to definitively characterize the tumor and TME features underlying the divergent clinical outcomes between HER2-low and HER2-0 TNBCs.

Here, we integrated data from an institutional retrospective cohort study and publicly available cohorts to determine and validate the effect of the HER2 expression level on the response to neoadjuvant immunotherapy in TNBC patients. We also leveraged single-cell RNA-sequencing (RNA-seq) and TCR-sequencing (TCR-seq) to characterize distinct baseline cellular and molecular patterns between two groups of 24 patients and characterize the dynamic changes in 22 patients treated with chemotherapy alone or in combination with immunotherapy (atezolizumab). Biological patterns were validated using an independent IMC dataset and several bulk transcriptome datasets. Our findings identified HER2 status as an independent biomarker for the response to neoadjuvant immunotherapy, supporting the reclassification of HER2-low TNBC as a distinct subtype to advance precision immunotherapy.

## Results

### Clinical cohort characteristics

**Fig. 1:**
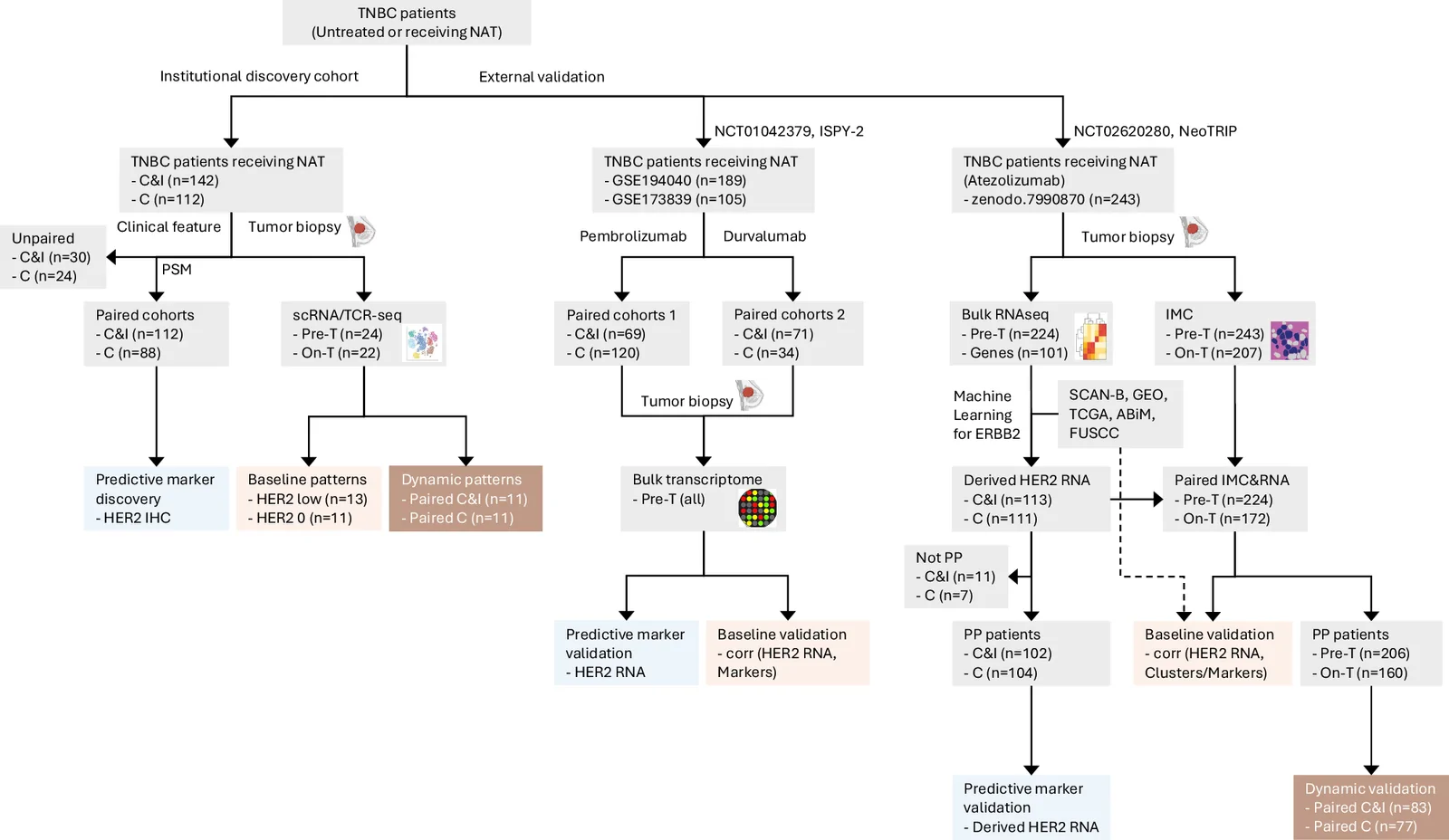
Overview of the cohorts and patients included in this study, exclusion criteria, laboratory tests, downstream analyses, and validation strategies. Further details are provided in the Methods section. TNBC triple-negative breast cancer, NAT neoadjuvant treatment, C chemotherapy, C&I Chemotherapy combined with immunotherapy, PSM propensity score matching, Pre-T pretreatment, On-T on-treatment, PP per protocol, IMC imaging mass cytometry, corr correlation.

To study the influence of HER2 IHC status (HER2-low vs HER2-0) on the response to immunotherapy response in TNBC patients, we retrospectively collected data of 254 TNBC patients, including 142 patients (83 HER2-low and 59 HER2-0) receiving immunotherapy in combination with chemotherapy (indicated as the immunotherapy below group for convenience) and 112 patients (62 HER2-low and 50 HER2-0) receiving chemotherapy only from 3 centers (Fig. 1). All individuals were female, with a median age of 45 years (IQR: 37–55 years). With respect to checkpoint inhibitor type, 131 patients (92.3%) received anti-PD-1 monoclonal antibodies, whereas 11 (7.7%) were treated with the anti-PD-L1 antibody atezolizumab (Supplementary Table 1). The overall pCR (pathological stage ypT0/Tis ypN0) rate was 48.0% (122/254) for all patients, with a pCR of 55.6% for the immunotherapy group and 38.4% for the chemotherapy group. A total of 24 pretreatment biopsy tissue samples (13 HER2-low and 11 HER2-0) were prospectively collected to study baseline differences and thereby identify the biological explanations that may be responsible for the different responses between HER2-low and HER2-0 patients receiving immunotherapy. Moreover, 11 matched post-immunotherapy and 11 post-chemotherapy biopsy tissues were collected to study the different changes in HER2-low and HER2-0 tissues after immunotherapy and chemotherapy (Fig. 1), respectively.

To validate the ability of HER2 expression to predict immunotherapy efficacy discovered in the institutional cohort, we further collected data from the NeoTRIP trial (NCT02620280) comparing nab-paclitaxel+carboplatin+atezolizumab (*n* = 102) with nab-paclitaxel+carboplatin (*n* = 104)^21^ and two treatment-paired (immunotherapy+chemotherapy vs. chemotherapy only) cohorts from the ISPY-2 trial (NCT01042379); paclitaxel+pembrolizumab (*n* = 69) was compared with paclitaxel alone (*n* = 120)^22^ in one cohort, and durvalumab/olaparib+control (*n* = 71) was compared with the control (*n* = 34)^23^ in the other cohort (Fig. 1). To validate the baseline biological differences between HER2-low and HER2-0 TNBC using data from the institutional single-cell cohort, imaging mass cytometry (IMC) data for TNBC from the NeoTRIP trial, tumor bulk microarray-transcriptomic data for TNBC biopsy tissue from the ISPY-2 trial (*n* = 189 and 105), and bulk RNA-seq transcriptomic data for TNBC tissue from SCAN-B (*n* = 874), FUSCC (*n* = 360), TCGA (*n* = 136), GEO (GSE163882, *n* = 90), and Abim (*n* = 65) were collected and analyzed (Fig. 1). Moreover, paired pretreatment and on-treatment IMC data from the NeoTRIP trial were collected and analyzed to validate the distinct dynamic responses after the initiation of immunotherapy revealed by the institutional single-cell data (Fig. 1).

### HER2-0 patients had a higher pCR rate to neoadjuvant immunotherapy than HER2-low patients did

**Fig. 2:**
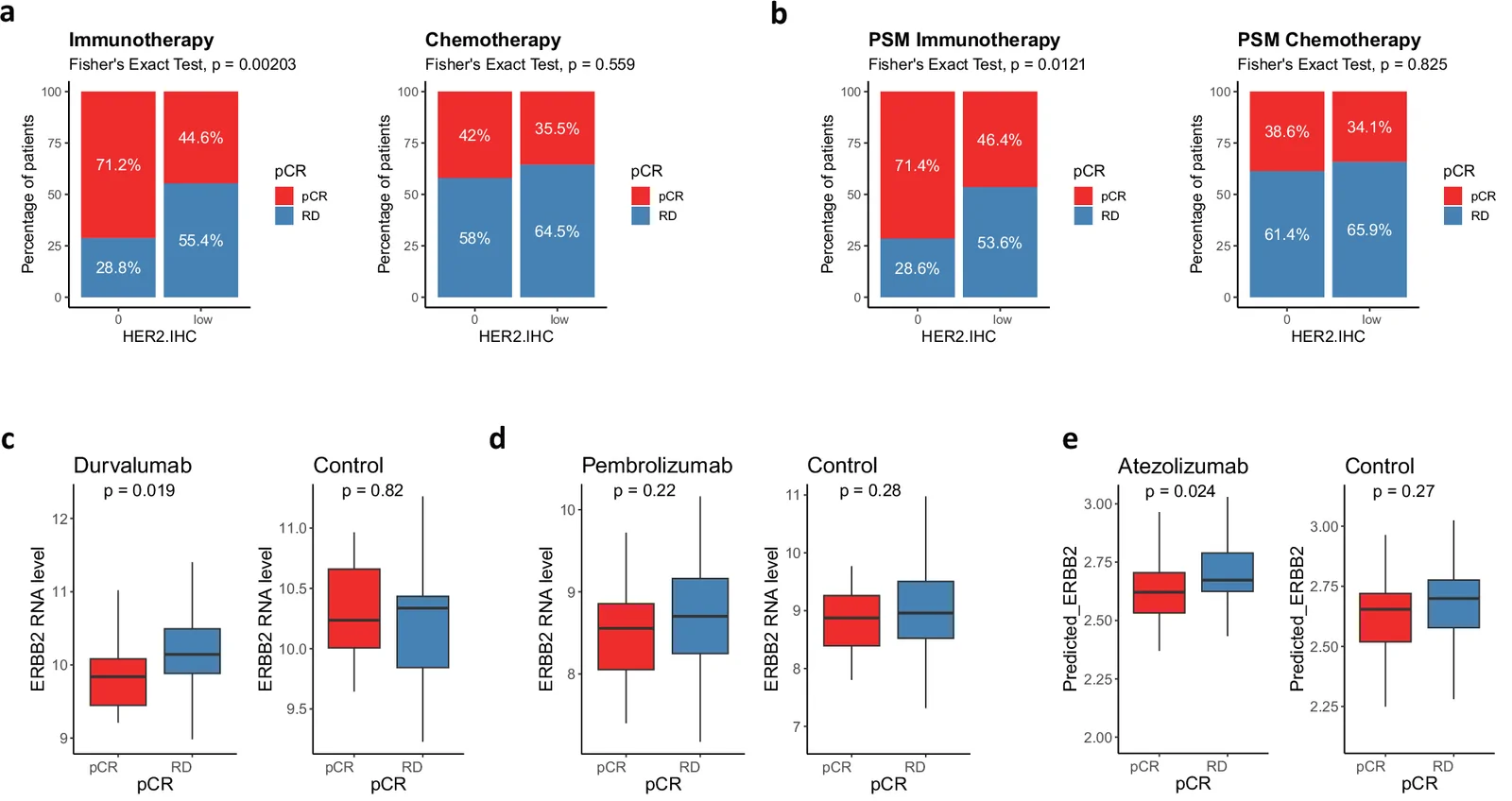
Effect of the HER2 expression level on the response to neoadjuvant immunotherapy. **a** Response to immunotherapy combined with chemotherapy (left) and chemotherapy only (right) according to HER2 status. **b** Response to immunotherapy combined with chemotherapy (left) and chemotherapy only (right) according to HER2 status after PSM. The missing PD-L1 test data were imputed by median. Stacked bar chart showing the percentage of patients who achieved a pathological complete response (pCR) versus those with residual disease (RD) after treatment, stratified by HER2 status. Statistical significance was assessed using Fisher’s exact test. Comparison of *ERBB2* RNA expression according to neoadjuvant response in the ISPY-2-durvalumab (GSE173839) (**c**), ISPY-2-pembrolizumab (GSE194040) (**d**), and NeoTRIP (**e**) trials. The box plots display the distribution of *ERBB2* expression in the pCR and RD groups. The centerline represents the median, the box limits represent the first and third quartiles (IQR), and the whiskers extend to the minimum and maximum values within 1.5 × IQR of the quartiles. The points beyond the whiskers are plotted individually as outliers. A Wilcoxon rank-sum test was used to calculate the *P* value shown. pCR pathological complete response, RD residual disease, PSM propensity score matching. IHC immunohistochemistry.

In the immunotherapy combined with chemotherapy group, compared with HER2-0 patients, HER2-low patients had a lower pCR rate (44.6% vs. 71.2%; OR 0.326; Fisher’s test *p* = 0.002); however, no significant difference was observed in the chemotherapy only group (35.5% vs. 42%; OR 0.760; Fisher’s test *p* = 0.559) (Fig. 2a). No differences in clinicopathological features were detected between HER2-low or HER2-0 patients in either the immunotherapy group or the chemotherapy group (Supplementary Table 1). To further avoid potential bias in the retrospective study, we performed propensity score matching (PSM) (based on age, grade, Ki67 expression, PD.L1.CPS score, cT stage, and cN stage) for the HER2-low and HER2-0 groups in the immunotherapy combined with chemotherapy and chemotherapy only groups to assess the influence of HER2 IHC on immunotherapy response in an unbiased manner. PD-L1 test was missing in 31.7% (45/142) immunotherapy patients and 58.93% (66/112) chemotherapy-only patients. Missing values for PD-L1 expression were imputed with the median. The results revealed that the HER2-0 group had a higher pCR rate than the HER2-low group did for immunotherapy combined with chemotherapy, not for chemotherapy alone (Fig. 2b). Sensitivity analyzes with different imputation strategies (mean or zero) and excluding patients with missing PD-L1 test remained consistent in both direction and statistical significance (Supplementary Fig. 1).

To demonstrate the generalizability of these findings, we sought to validate them using external datasets. No HER2 IHC data were publicly available from previously reported breast cancer neoadjuvant immunotherapy trials; thus, we looked for other proxies of HER2 levels for validation. We compared the *ERBB2* (HER2) RNA expression by HER2 status in 359 TNBC patients from the FUSCC dataset and revealed that HER2-low patients had significantly higher *ERBB2* (HER2) RNA expression levels (Supplementary Fig. 2a); thus, HER2 RNA expression data in the ISPY-2 trial were used instead. Compared with that in patients with a residual disease (RD), the *ERBB2* level in patients who achieved a pCR (Fig. 2c) was significantly lower for the durvalumab immunotherapy group (GSE173839); however, no significant difference was observed in the chemotherapy control group (Fig. 2c). In the pembrolizumab and paclitaxel group (GSE194040), *ERBB2* RNA levels were also lower in patients with pCR, although this trend was not statistically significant (Fig. 2d).

RNA expression data were also available from the NeoTRIP trial; however, the expression levels (FPKM values) of only 101 genes (not including *ERBB2*) were available. To estimate *ERBB2* expression levels, we constructed an ensemble machine learning model using the data for the 101 genes as inputs based on the SCAN-B dataset (874 TNBC patients)^24^ (Supplementary Fig. 2b). The predictive performance of the model was demonstrated to be robust in the external test datasets (Pearson’s *r* = 0.77 for the FUSCC-TNBC dataset^25–27^ (adjuvant surgery, *n* = 350), Pearson’s *r* = 0.57 for the GSE163882 dataset (neoadjuvant pretreatment biopsies, *n* = 90), Pearson’s *r* = 0.53 for the ABiM dataset (adjuvant surgery, *n* = 65), and Pearson’s *r* = 0.53 for the TCGA dataset (adjuvant surgery, *n* = 136) (Supplementary Fig. 2c–g). Thus, we used the predicted *ERBB2* RNA level as a proxy for HER2 status. After filtering per-protocol (PP) patients from the NeoTRIP trial, the analysis showed that the predicted *ERBB2* level of patients who achieved a pCR was significantly lower than that of patients with RD (Fig. 2e) in the atezolizumab immunotherapy group but not in the chemotherapy group (Fig. 2f). These findings corroborate that higher HER2 expression was a significant negative predictor of pCR among TNBC patients receiving immunotherapy combined with chemotherapy but not among those receiving chemotherapy alone.

### HER2-0 tumor cells showed higher expression of MHC and checkpoint molecules at baseline

**Fig. 3:**
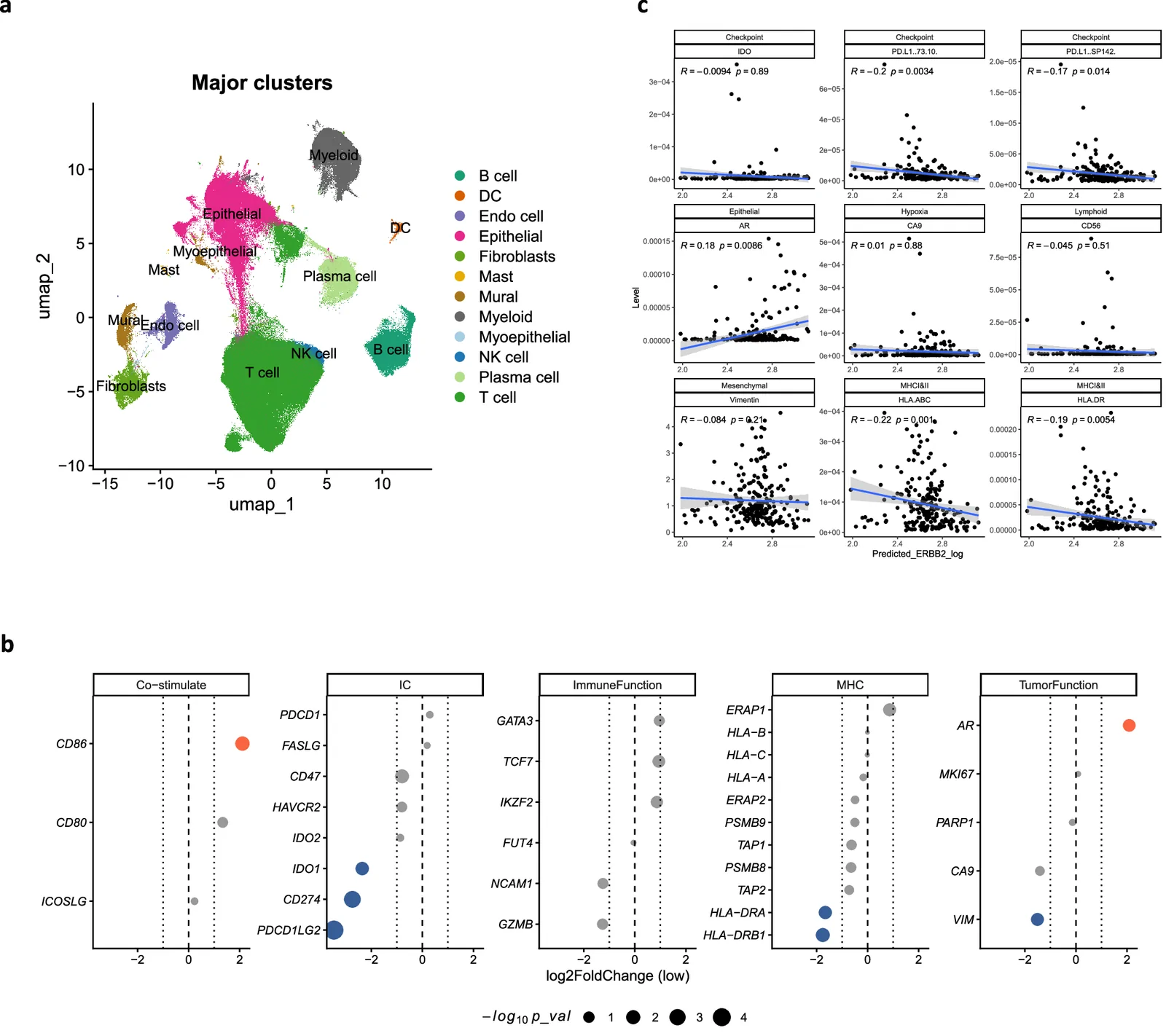
Differential gene expression patterns in tumor cells in patients with different HER2 levels. **a** Uniform manifold approximation and projection (UMAP) visualization of major cell types. **b** Dot plot showing the pseudobulk-based differentially expressed genes between HER2-low tumor cells and HER2-0 tumor cells at baseline. The genes are grouped by functional type. The *x*-axis represents the average log2-fold change in expression; positive values indicate higher expression in the HER2-low tumor cells. The size of each point corresponds to its statistical significance (-log10(p_val)). Genes are colored on the basis of their fold change in expression: markedly overexpressed (avg_log2FC ≥ 1, orange), markedly underexpressed (avg_log2FC ≤ -1, blue), or non-marked (gray). The dashed and dotted lines indicate the zero-fold change and the marked threshold, respectively. **c** Correlations between predicted *ERBB2* expression and the mean protein expression level in epithelial cells according to the IMC dataset. The proteins are labeled by functional type. Each point represents a patient sample in the scatter plot. A linear regression model (solid line) with a 95% confidence interval (shaded area) was fitted to the data. The strength and significance of the monotonic relationship were assessed using Pearson’s correlation coefficient (R) and the *P* value. The relationships between gene and protein markers (e.g., the *MKI67* gene and the Ki-67 protein) are detailed in Supplementary Table 2. DC dendritic cell, Endo cell endothelial cell, IC immune checkpoint, MHC major histocompatibility complex.

We used single-cell sequencing to investigate the underlying baseline and dynamic biological patterns underlying the ability of HER2 status to predict the response of TNBC to neoadjuvant immunotherapy. In total, 46 tumor biopsies were collected at baseline and 4 weeks after treatment initiation. The predictive ability of HER2 IHC data to predict the immunotherapy response for the single-cell cohort was similar to that for the whole cohort, indicating that this single-cell cohort was representative of the overall institutional clinical cohort (Supplementary Fig. 3a, b). After quality filtering, we obtained single-cell transcriptome data for 417,879 high-quality cells, including tumor cells (epithelial), stromal cells (mural cells, fibroblasts, endothelial cells, and myoepithelial cells), and immune cells (T cells, myeloid cells, NK cells, plasma cells, B cells, and mast cells) (Fig. 3a, and Supplementary Fig. 4a–c).

We first explored the differences in tumor cells between HER2-low and HER2-0 groups. Prior to treatment, *ERBB2* was more highly expressed in tumor cells in the HER2-low group than in the HER2-0 group (Supplementary Fig. 5), consistent with what was observed for HER2 protein expression according to IHC data. We then performed differential gene expression analyzes. Because immune checkpoint blockade (ICB) modulates interactions among immune and cancer cells via genes associated with immunogenicity^28,29^, we explored differences in the expression of genes that are associated with immunogenicity and were included in the IMC dataset (Supplementary Table 2), such as major histocompatibility complex (MHC)-related genes, immune checkpoint (IC)-related genes, tumor function-related genes, and immune function-related genes (Fig. 3b), between the two groups. We identified genes whose pseudobulk expression differed greatly (absolute average FC > 1) and found that tumor cells from HER2-0 patients expressed higher levels of immune checkpoint-related genes, such as *IDO1* (IDO), *CD274* (PD-L1), and *PDCD1LG2* (PD-L2); MHC-related genes, such as *HLA-DRA* and *HLA-DRB1* (MHC II); and EMT-related genes, such as *VIM* (vimentin). However, tumor cells from HER2-low patients expressed high levels of *AR* (related to poor immunotherapy response^30^) (Fig. 3b). The increased expression of immune checkpoint genes, MHC, and *VIM* in HER2-0 patients and of *AR* in HER2-low patients was validated using the IMC data in NeoTRIP, as the average PD-L1 (PD-L1 73.10 *p* = 0.0034; PD-L1, SP142 *p* = 0.014), HLA-ABC, and HLA-DR expression levels in tumor cells were significantly negatively correlated with the predicted *ERBB2* expression (lower *ERBB2* expression was associated with HER2-0 status, and higher *ERBB2* expression was associated with HER2-low status). IDO and vimentin expression showed similar trends, although the difference was not significant; AR protein expression was significantly positively correlated with the predicted *ERBB2* level (Fig. 3c). In conclusion, we validated that immune checkpoint gene and MHC levels are higher expressed in HER2-0 patients and that *AR* expression is higher expressed in HER2-low patients.

### HER2-0 immune cells exhibited a stronger tumor response and increased proliferation at baseline

**Fig. 4:**
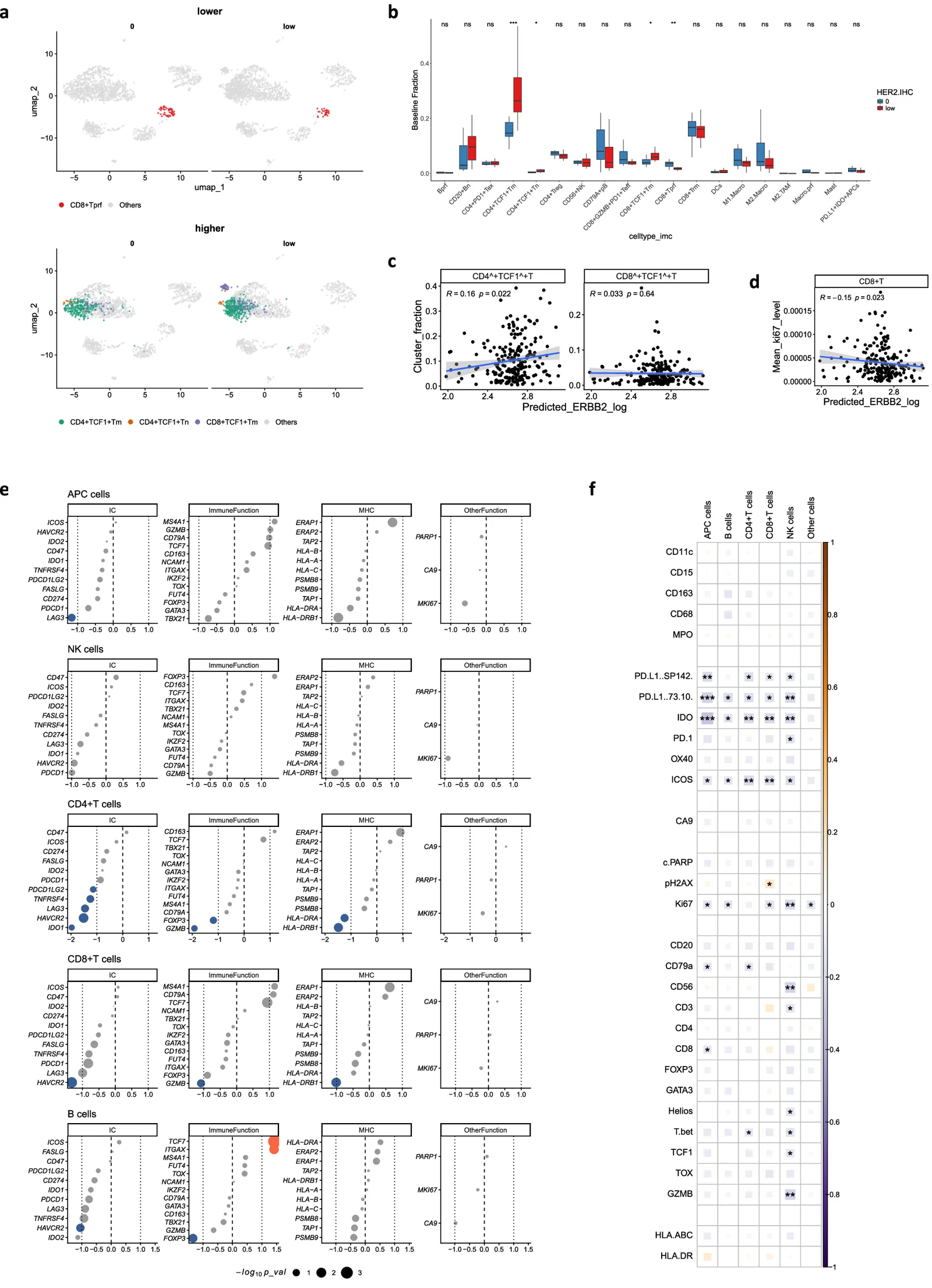
Differences in immune cells among patients with different HER2 expression levels. **a**, **b** Uniform manifold approximation and projection (UMAP) plots (**a**) and boxplots showing the difference in immune cell cluster proportions between HER2-low TNBC and HER2-0 TNBC (**b**). In the UMAP plots, 1500 cells were selected by random sampling for each group. In the boxplot, the centerline represents the median, the box limits represent the first and third quartiles (IQR), and the whiskers extend to the minimum and maximum values within 1.5 × IQR of the quartiles. The Wilcoxon rank-sum test was used to calculate the *P* values shown. The significance levels are indicated above the plots (ns: not significant; ^*^p < 0.05, ^**^p < 0.01, ^***^p < 0.001, and ^****^p < 0.0001). Correlations among predicted *ERBB2* expression, the proportions of CD4+TCF1+ T cells and CD8+TCF1+ T cells (**c**) and the cluster mean Ki67 protein expression level of CD8+ T cells (**d**) according to the IMC dataset. Each point represents a patient sample in the scatter plot. A linear regression model (solid line) with a 95% confidence interval (shaded area) was fitted to the data. The strength and significance of the monotonic relationship were assessed using Pearson’s correlation coefficient (*R*) and the *P* value. **e** Dot plot showing the pseudobulk-based differentially expressed genes between HER2-low immune cells and HER2-0 immune cells at baseline. Immune cells are grouped by cell type, and genes are grouped by functional type. The *x*-axis represents the average log2-fold change in expression; positive values indicate higher expression in HER2-low tumor cells. The size of each point corresponds to its statistical significance (-log10(p_val)). Genes are colored on the basis of their fold change in expression: markedly overexpressed (avg_log2FC ≥ 1, orange), markedly underexpressed (avg_log2FC ≤ -1, blue), or non-marked (gray). The dashed and dotted lines indicate the zero-fold change and marked threshold, respectively. **f** Correlation matrix between the mean protein expression level in each immune cell cluster and the predicted *ERBB2* level. Proteins are separated by functional category. The color and size of each square represent the strength and direction of the Spearman correlation coefficient, with blue indicating a negative correlation and orange indicating a positive correlation. The statistical significance of each correlation is denoted by asterisks: ^***^p < 0.001, ^**^p < 0.01, and ^*^p < 0.05. Nonsignificant correlations (*p* ≥ 0.05) are not marked. The relationships between gene and protein markers (e.g., *MKI67* genes and Ki-67 proteins) are detailed in Supplementary Table 2. IC immune checkpoint, MHC major histocompatibility complex.

No significant differences in the proportion of major immune cell clusters in tumor microenvironment (TME) were observed between the pretreated HER2-low and HER2-0 pretreatment groups (Supplementary Fig. 6). Thus, we performed a second round of cluster analysis of immune cells (Supplementary Fig. 7a). Forty-four clusters were generated and annotated with general cell function markers and markers in the IMC dataset for collaborative analysis and validation, and 19 cell types were ultimately identified (Supplementary Fig. 7b–d). Compared with HER2-0 patients, HER2-low patients had more CD4+TCF1+ T exhausted and naive cells and more CD8+TCF1+ Tm cells. Moreover, there was a lower proportion of proliferating CD8+ T cells in HER2-low patients (Fig. 4a, b). The difference in CD4+TCF+ T cell clusters was validated using the IMC dataset; however, there was no significant difference observed for CD8+TCF1+ T cells (Fig. 4c). In the absence of an annotated proliferating CD8+ T cell cluster in the IMC dataset, we assessed the mean Ki67 expression level within CD8+ T cells as a surrogate metric to validate their higher proportion in HER2-0 patients. The results revealed that the Ki67 level was negatively correlated with the predicted *ERBB2* level (Fig. 4d), which is consistent with the findings obtained for our institutional single-cell transcriptome dataset.

We then performed pseudopulk-based differential gene expression analysis among various cell types (CD4+ T cells, CD8+ T cells, NK cells, B cells, APCs (DCs and macrophages), and mast cells) to investigate differences in functional status among immune cells between the two groups. In general, the expression of immune checkpoint genes such as PD-L1 (*CD274*), PD-1 (*PDCD1*), HAVCR2 (*TIM-3*), and *LAG3* were higher expressed in immune cells from HER2-0 patients (Fig. 4e). The general exhaustion features of HER2-0 immune cells implied their tumor reactivity, as previous studies have shown that tumor-reactive immune cells exhibit an exhausted phenotype due to persistent tumor antigen stimulation^31–34^. Immune checkpoint expression levels were validated to be generally negatively correlated with predicted HER2 RNA levels for the IMC dataset (Fig. 4f). Additionally, Ki67 (*MKI67*) expression was generally higher in HER2-0 patients in both the institutional dataset and the IMC dataset (Fig. 4e, f). Notably, *GZMB* was also significantly highly expressed in both CD4+ and CD8+ T cells in HER2-0 patients, with a similar trend observed for the IMC dataset (Fig. 4e, f); this indicates a stronger tumor response and a potentially better response to immunotherapy in HER2-0 patients.

Furthermore, we performed spatial analysis to evaluate the interactions between epithelial cells and immune cells according to IMC data. The interaction indices of Tregs, CD8+GZMB+ T cells, PD-L1+IDO+ APCs and PD-L1+ APCs with epithelial cells were significantly negatively correlated with predicted *ERBB2* expression (Supplementary Fig. 8). These findings are consistent with the higher response rate of HER2-0 patients to immune checkpoint inhibitor treatment. In conclusion, compared with those in HER2-low patients, immune cells in HER2-0 patients exhibited higher proliferation (indicated by increased Ki67 levels), a stronger tumor response (indicated by increased *GZMB* levels and a higher PD-L1+APC-to-epithelial cell spatial interaction index), and an exhaustion phenotype (indicated by increased checkpoint expression) as feedback mechanisms.

### Validation of baseline gene expression patterns with multicohort integrated bulk RNA datasets

**Fig. 5:**
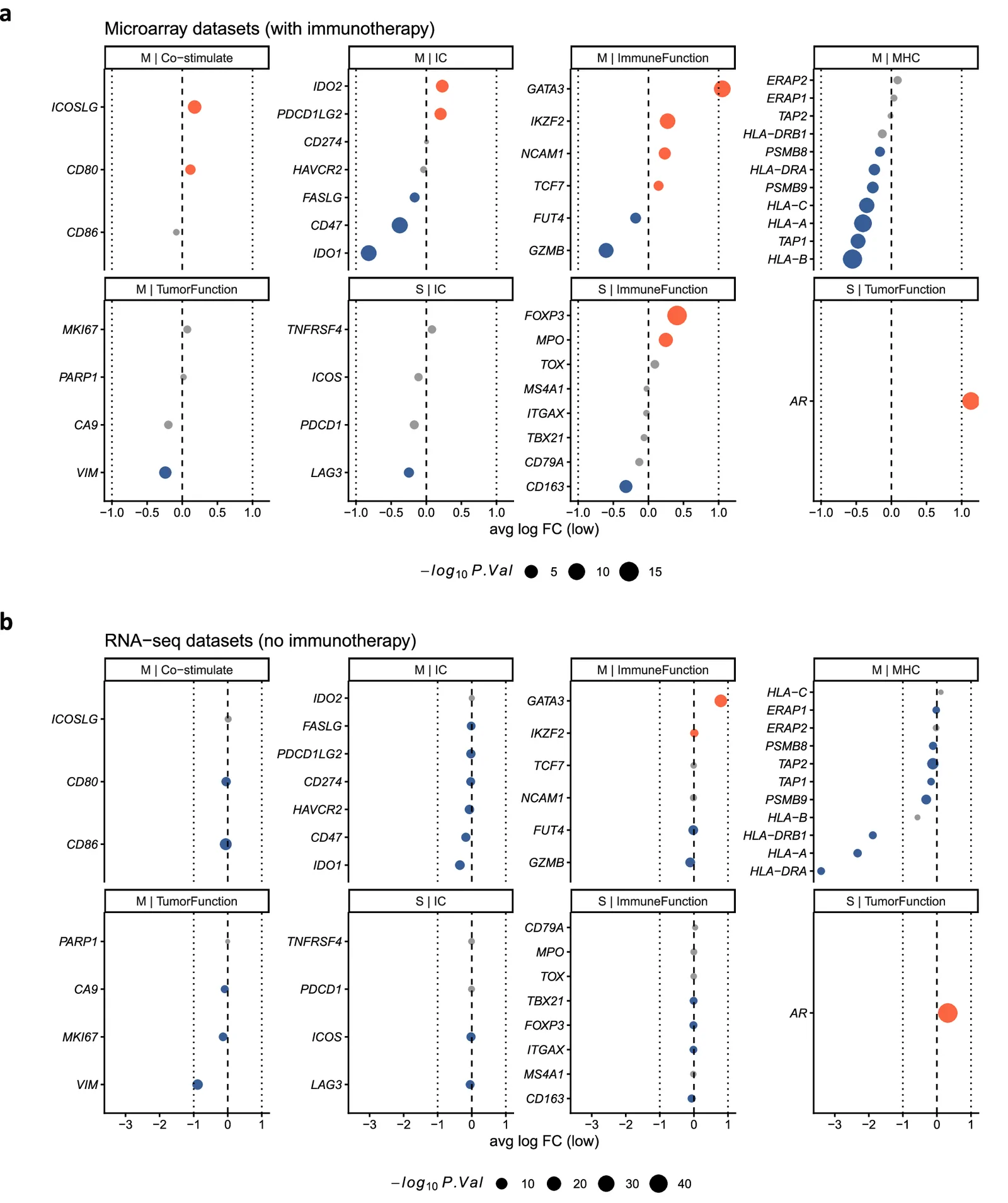
Validation of baseline gene markers in bulk RNA datasets. Dot plot showing the differentially expressed genes between HER2-low TNBC and HER2-0 TNBC at baseline in the microarray datasets with immunotherapy (**a**) and in the RNA-seq datasets without immunotherapy (**b**). Genes are grouped by orientation type (M: multiple cell types shared; S: cell-type specific) and functional type. The *x*-axis represents the average log2-fold change in expression; positive values indicate higher expression in HER2-low tumor cells. The size of each point corresponds to its statistical significance (-log10(p_val)). Genes are colored on the basis of their fold change in expression: overexpressed (avg_log2FC > 0, orange), underexpressed (avg_log2FC < 0, blue), non-significant (*P* value >= 0.05, gray). The dashed and dotted lines indicate the zero-fold change and marked threshold, respectively. IC immune checkpoint, MHC major histocompatibility complex.

We next sought to generalize the findings regarding baseline biological patterns derived from single-cell RNA/protein datasets to larger-scale bulk RNA datasets. Because gene counts were obtained for all cells in the bulk-RNA dataset, we classified markers into epithelial-specific, immune cell-specific, and multiple-cell-type markers to determine the source cell types (Fig. 5a, b). Specifically, the increased expression of cell-type-specific markers in the bulk RNA-seq dataset could be explained by increased expression of these markers in the corresponding cell type. *LAG3* was confirmed to be more highly expressed in patients with lower *ERBB2* expression, and the expression of *ICOS* differed significantly according to the RNA-seq datasets (Fig. 5a, b). AR expression was significantly positively correlated with *ERBB2* expression (Fig. 5a, b). These findings for cell type-specific markers mostly correspond to the patterns observed in the single-cell data.

Regarding markers shared among multiple cell types, the higher expression of MHCs in patients with lower *ERBB2* RNA expression was consistent with the high expression of MHCs in the epithelial and immune cells of HER2-0 patients (Fig. 5a, b). The levels of immune checkpoints expressed in multiple cell types, such as *IDO1* (much more important than *IDO2*), *CD47*, *FASLG*, and *CD274* (only in the RNA-seq datasets), were negatively correlated with *ERBB2* expression, consistent with the higher expression of immune checkpoints (ICs) in HER2-0 tumor and immune cells (Fig. 5a, b). Higher *GZMB* expression was also observed in patients with lower *ERBB2* expression, consistent with the higher expression of *GZMB* in T cells in HER2-0 patients (Fig. 5a, b). *TCF7* expression was positively correlated with *ERBB2* expression according to the microarray dataset but not significantly correlated with *ERBB2* expression according to the RNA-seq dataset (Fig. 5a, b). *VIM* expression was negatively correlated with *ERBB2* expression, consistent with the lower expression of *VIM* in HER2-low epithelial cells (Fig. 5a, b). *MKI67* expression was slightly negatively correlated with *ERBB2* expression according to the RNA-seq dataset, partly confirming that Ki67 expression was higher in HER2-0 immune cells (Fig. 5a, b). In general, the differences in bulk RNA profiles between HER2-0 and HER2-low patients are well consistent with the obtained single-cell profiles.

### Checkpoint expression decreased and T cell clonal expansion occurred after immunotherapy in HER2-0 patients

**Fig. 6:**
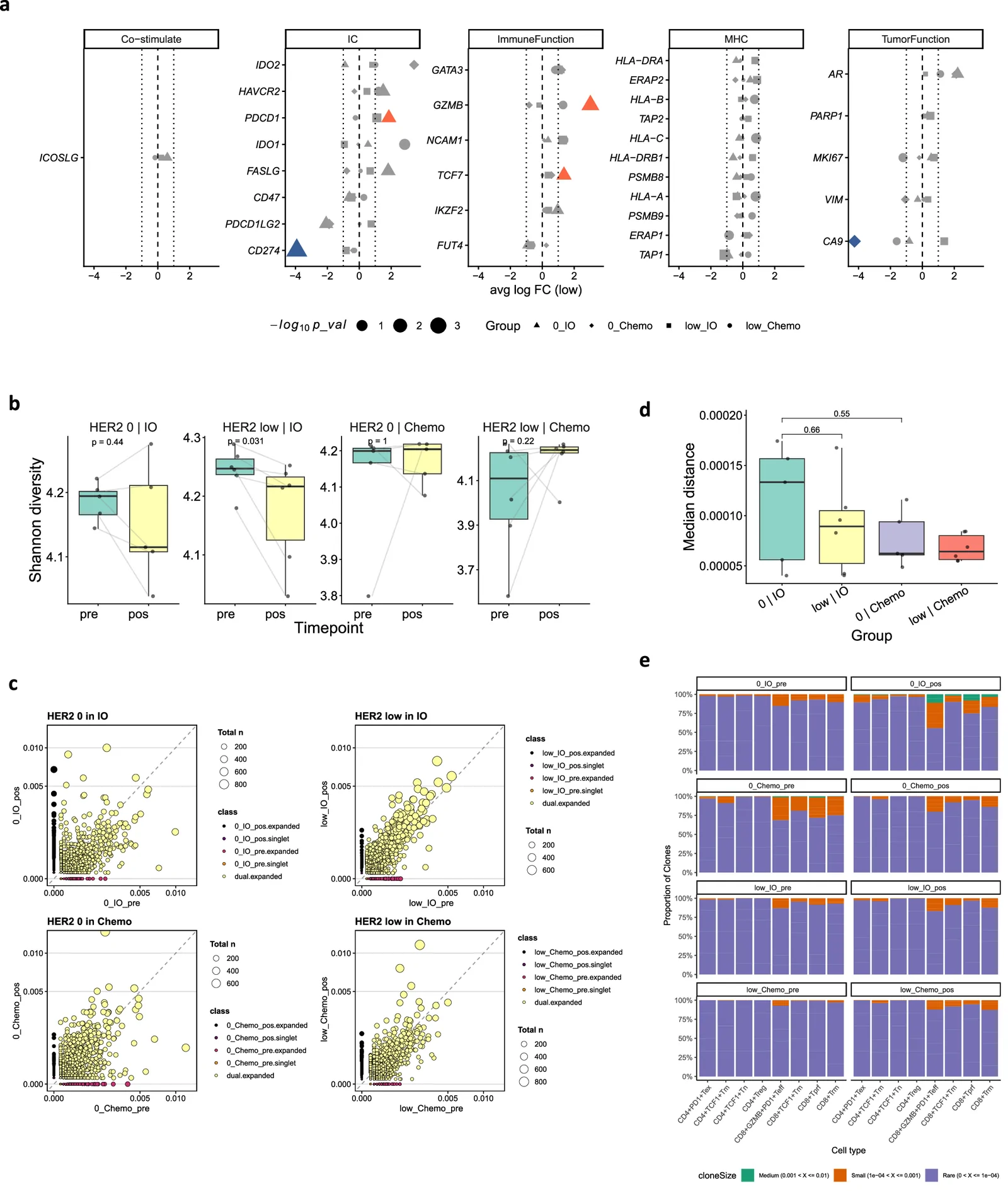
Dynamic changes in tumor and immune cells following immunotherapy. **a** Dot plot showing the pseudobulk-based differentially expressed genes in tumor cells between baseline and on-treatment samples in the single cell dataset. Genes are grouped by functional type. Groups are labeled with various shapes. The *x*-axis represents the average log2-fold change in expression; positive values indicate higher expression in HER2-low tumor cells. The size of each point corresponds to its statistical significance (-log10(p_val)). Genes are colored on the basis of their fold change in expresion: markedly overexpressed (avg_log2FC ≥ 1, orange), markedly underexpressed (avg_log2FC ≤ -1, blue), or non-marked (gray). The dashed and dotted lines indicate the zero-fold change and marked threshold, respectively. **b** Shannon diversity of TCR repertoires across treatment groups. Each point represents an individual patient, with paired samples connected by lines. Boxplots show median and interquartile range. Comparisons between timepoints were performed using two-sided Wilcoxon signed-rank tests, with *p*-values indicated. **c** Scatter plot comparing the proportional abundance of T-cell clones (based on TCR gene sequences) in the pretreatment samples (*x*-axis) to that in the on-treatment samples (*y*-axis). Each point represents a unique T-cell clone. The size of the point corresponds to the total abundance of that clone across the group. Clones on the diagonal (dashed line) were equal in abundance before and after treatment, whereas clones off the diagonal are those that exhibited expansion (above the line) or contraction (below the line). **d** Clone-level redistribution of TCR repertoires quantified by median distance from the diagonal following treatment. Each point represents the median absolute distance across all clones of one patient. Boxplots show median and interquartile range. Statistical comparisons between groups were performed using two-sided Wilcoxon rank-sum tests, with *p*-values indicated. **e** Clonal expansion landscape of T cell types across patient groups. Stacked bar charts showing the proportional distribution of T-cell clones (*y*-axis), categorized by their size (cloneSize), for different T-cell types (*x*-axis) in each patient group. The interior color indicates the relative abundance of each clone (e.g., rare, small, and medium). IC immune checkpoint, MHC major histocompatibility complex, IO immunotherapy, Chemo chemotherapy.

We then analyzed the paired pretreatment and on-treatment single-cell data to explore the dynamic changes in HER2-0 patients and HER2-low patients in response to immunotherapy. With respect to the dynamic changes of pseudobulk gene expression in tumor cells, after anti-PD-L1 immunotherapy, *CD274* (PD-L1) levels significantly decreased in HER2-0 patients, while no significant difference in PD-L1 expression was observed in HER2-low patients. Moreover, after immunotherapy, *GZMB* expression increased markedly in HER2-0 patients, which may cause the increase in *PDCD1* (PD-1) expression as a feedback mechanism^34^ (Fig. 6a). In HER2-low patients, no significant changes of epithelial gene expressions were observed after anti-PD-L1 immunotherapy (Fig. 6a). The changes in molecular expression levels in epithelial cells after immunotherapy were not significantly different between HER2-0 and HER2-low patients according to the IMC dataset (Supplementary Fig. 9).

We then explored the dynamic changes in immune cells. No significant changes were observed in immune cell cluster fractions in HER2-low or HER2-0 patients after immunotherapy (Supplementary Fig. 10a) or according to the IMC dataset (Supplementary Fig. 10b). Thus, we performed pseudobulk-based DEG analysis and found that both HER2-low patients and HER2-0 patients showed a decrease in *CD274* (PD-L1) expression in lymphoid cell clusters after anti-PD-L1 treatment. The expression of *GZMB* generally decreased in immune cell clusters in HER2-low patients after immunotherapy (Supplementary Fig. 11a). This trend is consistent with what was observed for the IMC data, which showed that the change of PD-L1 expression was generally positively correlated with the predicted *ERBB2* level, whereas the change of *GZMB* expression was generally negatively correlated with the predicted *ERBB2* level (Supplementary Fig. 11b).

We further performed TCR sequencing analysis to directly assess dynamic changes in T cells before and after treatment. We first assessed global TCR repertoire diversity using Shannon diversity. A modest decrease in diversity was observed in HER2-0 and HER2-low patients receiving immunotherapy, while a modest increase was observed in patients receiving chemotherapy only (Fig. 6b), suggesting the expansion of a limited number of dominant T cell clones during immunotherapy. To more sensitively capture clonal dynamics, we then inspected clone-level changes. HER2-0 patients exhibited a large increase in T cell clonal expansion after immunotherapy, with more data points being located away from the diagonal (Fig. 6c). We next quantified clone-level changes in TCR abundance by measuring the distance of each clone from the diagonal (*y* = *x*). This metric reflects the magnitude of clonal expansion or contraction independent of global diversity. Notably, HER2-0 patients receiving immunotherapy exhibited higher median distances compared with HER2-low patients, indicating increased clonal redistribution (Fig. 6d). In contrast, both HER2-0 and HER2-low patients receiving chemotherapy showed lower levels of clonal change (Fig. 6d). These findings suggest that immunotherapy induces more pronounced clonal remodeling in HER2-0 tumors. By combining the TCR data and cell annotation data, we next investigated the clone size distribution by cell type. CD8+GZMB+PD1+ Teff cell and CD8+ Tprf cells exhibited more clonal expansion in HER2 0 patients than in HER2-low patients after immunotherapy (Fig. 6e). These findings indicate that T cells were antigen-experienced in HER2-0 patients and are consistent with the higher response rate of these patients to immunotherapy. In conclusion, HER2-0 patients exhibited significantly decreased PD-L1 expression in epithelial cells after anti-PD-L1 treatment, whereas HER2-low patients showed no significant decrease in PD-L1 expression in epithelial cells but showed decreased *GZMB* expression in immune cells. Moreover, modest CD8+GZMB+PD1+ Teff cell and CD8+ Tprf cell clonal expansion was observed in HER2-0 patients after immunotherapy, and this expansion may have been key to the response of HER2-0 patients to immunotherapy.

## Discussion

In this study, through the use of data from multiple cohorts, we validated the negative predictive efficacy of HER2 expression status for a pCR following neoadjuvant immunotherapy combined with chemotherapy but not chemotherapy alone in TNBC patients. Single-cell omics data revealed that HER2-0 patients generally exhibited higher baseline MHC and immune checkpoint (IC) levels in both tumor and immune cells and a greater abundance of Tprf cell subsets, indicating a potentially stronger immune response. Furthermore, after anti-PD-L1 treatment, HER2-0 patients exhibited an overall reduction in PD-L1 expression in tumor cells and modest clonal expansion of the Teff and Tprf subsets, effects that were not significant in HER2-low patients, suggesting a greater treatment response.

From a clinical perspective, we first systematically studied the effect of HER2 status on the response of TNBC to immunotherapy. We proved that HER2 status is an independent negative predictor of immunotherapy efficacy by performing PSM of potential biological factors such as PD-L1 status and the Ki67 index. We further validated these findings using three other publicly available datasets using HER2 RNA expression as a proxy of HER2 IHC levels, though the correlation between HER2 IHC status and *ERBB2* RNA expression is imperfect and the machine learning–based estimation of *ERBB2* expression in the NeoTRIP cohort introduces additional uncertainty, the consistency in results direction in all datasets and statistical significance in two datasets increases the robustness of our conclusions. Notably, the result is not significant in I-SPY2 pembrolizumab. This may reflect insufficient proxy accuracy of *ERBB2* RNA expression or relatively small cohort sample size (*n* = 69); however, the possibility that this represents a true null finding or reflects therapeutic heterogeneity cannot be excluded. For comparison, we also included paired chemotherapy-only cohorts, and the nonsignificant difference observed for these cohorts was consistent with the results of a previous pooled analysis in which no significant difference in the pCR rate was observed between patients with HER2-low and HER2-0 TNBC after chemotherapy^15^. These results indicate that HER2 status has an important influence on the response to neoadjuvant immunotherapy. In future studies on neoadjuvant immunotherapy for TNBC, HER2 status should be included as an important consideration, and approaches to overcome the insensitivity of patients with HER2-low status to immunotherapy need to be developed. Moreover, in combination with previous findings that HER2-low patients exhibit differences in outcomes following traditional non-immunotherapy neoadjuvants^15,16^, responses to ADC drugs^13,14^, and PFS following CDK4/6 inhibitor treatment^35^ compared with HER2-0 patients, the results of our study on immunotherapy further highlight the need to consider HER2-low TNBC as a separate subtype of HER2-negative TNBC for precision cancer treatment.

Biologically, MHC molecules can present tumor antigens to T cells, playing a pivotal role in initiating anticancer immunity, and the downregulation of MHC-I expression has been described as a mechanism of intrinsic and acquired resistance to immunotherapy^36,37^. These previous reports are consistent with our findings that immunotherapy-insensitive HER2-low TNBC tissues exhibit lower expression of MHC molecules in both tumor and immune cell clusters, which is consistent with past work linking the expression of MHCII by cancer cells to the response to neoadjuvant ICB^38^. High MHC levels and corresponding high tumor antigen levels can increase the number of tumor-reactive immune cells; thus, immune cells generally exhibit an exhaustion phenotype as a feedback mechanism^31–34^, consistent with the high levels of immune checkpoints (ICs) observed in HER2-0 TNBC in this study. Moreover, the elevated Ki67 expression in HER2-0 TNBC T cells in our study is consistent with the established role of T cell proliferation in driving response to ICB^21^. Regarding T cell dynamics, the ultimate amplitude and quality of the response, which is initiated through MHC-restricted antigen recognition by the T cell receptor, is regulated by a balance between costimulatory and inhibitory signals (that is, immune checkpoints)^39^. Thus, the increased T cell clonal expansion after the downregulation of PD-L1 by anti-PD-L1 treatment observed in our study is reasonable because of the higher baseline MHC, PD-L1, and T cell Ki67 levels. Therefore, high expression of MHC and ICs and high levels of T cell proliferation are key to the response of HER2-0 TNBC to neoadjuvant immunotherapy. This insight can guide future research in breast cancer immunotherapy.

Mechanistically, recent work demonstrates that HER2-low TNBC exhibits an immune-evasive phenotype characterized by epigenetic silencing of antigen presentation and immune-related pathways, including hypermethylation of *HLA* genes and reduced immune cell infiltration^40^. Our findings are highly consistent with these observations, as we identify reduced expression of MHC and immune checkpoint molecules, together with diminished T cell activity, in HER2-low tumors at single-cell resolution. Importantly, our study extends these molecular insights by demonstrating that such immune contexture differences translate into clinically meaningful variation in response to neoadjuvant immunotherapy across multi-center cohorts, and further delineates their dynamic evolution during treatment. Beyond epigenetic regulation, *ERBB2* signaling has been shown to suppress MHC-I expression and promote immune evasion through downstream signaling pathways, with *ERBB2* inhibition restoring antigen presentation and enhancing immunotherapy response in small cell lung cancer experimental models^41^. While these studies provide compelling evidence for a causal role of *ERBB2* signaling in regulating tumor immunogenicity, they are derived from controlled experimental systems and distinct tumor types. In contrast, our study is observational in nature and does not directly interrogate causality. Therefore, HER2 expression in TNBC should be interpreted primarily as a biomarker associated with differential TME and immunotherapy response, rather than a definitive mechanistic driver. Additionally, our analysis revealed an upregulation of androgen receptor (*AR*) expression in baseline HER2-low tumors. *AR* expression is a key feature of the luminal androgen receptor (LAR) subtype of TNBC^42^, which is often characterized by a more immune-excluded phenotype with diminished immune activation^43^. Our findings suggest that the high *AR* expression may contribute to immune suppression in HER2-low TNBC, providing a potential mechanism by which these tumors are less responsive to immunotherapy. Collectively, these findings support a model in which HER2-low status reflects the convergence of multiple underlying biological processes, including epigenetic regulation, oncogenic signaling, and *AR* upregulation, that together shape tumor immunogenicity and therapeutic sensitivity. This integrated perspective provides a conceptual framework for understanding HER2-low TNBC as a biologically distinct entity and highlights the need for future mechanistic and interventional studies to disentangle the relative contributions and causal relationships of these processes.

The limitations of our study include a relatively small sample size for single-cell analyzes. While the clinical associations were validated in both anti-PD-1 and anti-PD-L1 cohorts, and the intrinsic immune differences between the two HER2‑expression groups were robustly characterized by institutional analysis and datasets validation, it was noted that the dynamic single‑cell analyzes were derived exclusively from anti‑PD‑L1‑treated patients due to data availability. Future prospective cohorts with larger sample sizes and including single‑cell or spatial transcriptomic data from patients treated with different types of immune checkpoint inhibitors are needed to robustly confirm the dynamic immune patterns. Additionally, the IMC dataset analyzed in our study does not provide direct HER2 IHC or *ERBB2* bulk RNA expression information. Though the robust performance of our predictive model for *ERBB2* expression and the large size of the IMC dataset increase the confidence of validations, the use of predicted *ERBB2* expression in the IMC dataset does not constitute fully independent validation and future studies incorporating large cohort single-cell or spatial datasets with directly measured HER2 IHC status will be important to further strengthen these findings.

In conclusion, our study establishes HER2 expression as a significant negative predictor of pCR in TNBC patients receiving neoadjuvant immunotherapy. This finding supports the reclassification of HER2-low TNBC as a distinct subtype to refine precision immunotherapy strategies. In future studies of neoadjuvant immunotherapy for TNBC, HER2 status should be strongly considered, and approaches to overcome the insensitivity of HER2-low patients to immunotherapy need to be developed.

## Methods

### Institutional retrospective cohort design

This multicenter, retrospective, real-world study (RWS) included patients with early-stage TNBC who received neoadjuvant immunotherapy between October 2019 and December 2024. The participants were recruited from three institutions: the National Cancer Center/Cancer Hospital, Chinese Academy of Medical Sciences and Peking Union Medical College; the Cancer Hospital of HuanXing ChaoYang District; and Sanhuan Cancer Hospital in Beijing. The study protocol was conducted in accordance with the principles of the Declaration of Helsinki.

The patient inclusion criteria were as follows: (1) female, aged ≥ 18 years; (2) newly diagnosed, previously untreated, early-stage TNBC; (3) comprehensive baseline data and at least one posttreatment radiological assessment in accordance with the Response Evaluation Criteria in Solid Tumors (RECIST) version 1.1^44^; and (4) received neoadjuvant therapy with programmed cell death protein-1 (PD-1)/programmed cell death ligand-1 (PD-L1) inhibitors combined with chemotherapy or chemotherapy only after clinical assessment by an attending physician. The key exclusion criteria were as follows: any history of autoimmune disease; acute infection; or recent use of systemic corticosteroids, immunostimulatory agents, or immunosuppressive agents. Patients received neoadjuvant chemotherapy therapy (NACT) and immunotherapy drugs according to their clinical routine.

The following demographic and treatment-related information of the patients was retrospectively collected: sex, age, tumor size, lymph node involvement, clinical stage (according to the 8th edition of the AJCC on Cancer), histopathological type, immunohistochemical marker levels, and details regarding immunotherapy and chemotherapy administered. Moreover, the efficacy of the neoadjuvant treatment, achievement of pCR (primary endpoint, defined as ypT0/Tis and ypN0), and adverse events (AEs) were recorded. The collected information was derived from electronic medical records and documented using structured data forms.

This study was approved by the Ethics Committee of the National Cancer Center/Cancer Hospital, Chinese Academy of Medical Sciences (No. 18–216/1794), and complied with all relevant ethical regulations. All patients provided written informed consent for the collection of tissue and blood samples for research and genomic profiling. Informed consent was waived for retrospective cohort analyzes because of the use of deidentified data and minimal risk to participants.

### Bulk transcriptome data collection and preprocessing

To assess the predictive value of *ERBB2* RNA expression for the immunotherapy response, we used data in gNormalizedSignal (log2) format in the GSE194040 and GSE173839 datasets from the ISPY-2 trial. The GSE194040 dataset included data from 69 patients who received paclitaxel+pembrolizumab and 120 patients who received paclitaxel^22^. The GSE173839 dataset included data for 71 patients who received control + durvalumab/olaparib (*n* = 71) and 34 patients who received control treatment^23^.

Regarding the ability of expression data for other genes to be used to predict *ERBB2* RNA expression, because the RNA-seq data included in the NeoTRIP dataset were in FPKM (log2) format, we used log2 FPKM/TPM values (FPKM values preferentially) for model construction and evaluation. The FPKM values for 101 genes were obtained from the supplementary tables supplied in the original article reporting the IMC data of the NeoTRIP trial^21^. Data from the SCAN-B, ABiM, FUSCC, TCGA, and GEO (GSE163882) datasets were also included; TNBC patients were filtered for model construction and external tests. Specifically, for TNBC patient included in the SCAN-B (*n* = 874) and ABiM (*n* = 65) datasets, we obtained FPKM data from the Mendeley Data repository^45^. For TNBC patients included in the FUSCC dataset (*n* = 360), we obtained TPM data (no FPKM data were provided) from the Fudan Data Portal (https://data.3steps.cn/cdataportal/). For TNBC patients included in the TCGA dataset (*n* = 136), we obtained FPKM data from the TCGAbiolinks package in R. For TNBC patients included in the GEO (GSE163882) dataset (*n* = 90), we obtained TPM data (no FPKM data were provided) from the GEO dataset. Log2 transformation of TPM/FPKM data was then performed.

### Machine learning model for predicting *ERBB2* RNA expression

We used FPKM-normalized RNA-seq data from 874 TNBC patients included in the SCAN-B dataset to construct the model (Supplementary Fig. 1b). Expression data for a total of 95/101 genes whose expression levels were evaluated in the NeoTRIP trial were included in the SCAN-B dataset and were used as model inputs. Data for all 95 of these genes were included in the other test datasets. For feature engineering, all the input and target features were log2 normalized (log2(*x* + 1)). Models such as generalized linear model with elastic net regularization (glmnet), ranger (random forest), SVM, extreme gradient boosting (xgboost), and Bayesian additive regression tree (Bart) were evaluated. The hyperparameters of the models were tuned via tenfold cross-validation with *R*-square evaluation metrics (regr.rsq in mlr3). For glmnet, the hyperparameter set was alpha (0, 0.1, 0.2, 0.4, 0.5, 0.6, 0.8, and 1) and s (0.0001, 0.001, 0.01, 0.1, and 1); for ranger, the hyperparameter set was mtrys (3, 6, 10, 40, 60, 80, and 95), num.treess (50, 100, 500, 700, and 1000), and min.node.sizes (1, 3, 5, 7, 10, 15, and 20); for xgboost, the hyperparameters were eta (0.01, 0.1, 0.3, 0.5, 0.7, and 1), max_depth (3, 6, 10, 15, and 20), subsample (0.5, 0.75, and 1), colsample_bytree (0.5, 0.75, and 1), and lambda (0.0001, 0.001, 0.01, 0.1, 10, 100, and 1000); and for Bart, the hyperparameters were ntree (50, 100, 200, 300, 400, and 500), k (1, 2, 3, 4, and 5), power (1.5, 2, 2.5, and 3), and base (0.8, 0.85, 0.9, 0.95, and 0.99). Then, we ensembled all the tuned models and retrained them using the SCAN-B TNBC dataset to obtain the final predictive model for application. To validate model externally, we used TNBC data from the FUSCC dataset (*n* = 360), TNBC data from the TCGA dataset (*n* = 136), data for TNBC biopsies collected before treatment from the GEO dataset (GSE163882) (*n* = 90), and TNBC data from the ABiM dataset (*n* = 65). RNA-seq data in FPKM/TPM format (FPKM values preferred) were used. To evaluate performance, Pearson’s r was used because we focused on the relative rank of *ERBB2* levels across patients. Models were built via the R mlr3 framework.

### Human sample collection and processing for single-cell sequencing

In this study, biopsy tissues were obtained from a total of 24 patients receiving neoadjuvant therapy for single-cell sequencing; all of these patients were enrolled in our previous study^46,47^. A total of 12 patients received chemotherapy only, and 12 received combination therapy (chemotherapy plus atezolizumab). PD-L1 levels were not used for patient screening in our previous studies. The patients were treated in accordance with routine clinical protocols.

The samples were derived from primary breast tumors or local lymph nodes. For tumor biopsy samples, pre- and posttreatment samples were obtained at a baseline of <2 weeks prior to treatment and 4 weeks after treatment initiation. Each tumor was punctured by two or three needles. All sample collection procedures complied with routine clinical practice. After collection, the tumor biopsy samples were first stored in RNAlater RNA stabilization reagent (QIAGEN) and kept on ice to avoid RNA degradation. Biopsy samples from each tumor at each time point were then combined before tissue dissociation. The samples were placed in RPMI-1640 medium (Gibco) and enzymatically digested with a gentle MACS Tumor Dissociation Kit (Miltenyi Biotec) for 60 min on a rotor at 37 °C in accordance with the manufacturer’s protocol. The dissociated cells were subsequently passed through a 40-μm cell strainer (BD) in RPMI-1640 medium (Invitrogen) supplemented with 10% FBS until uniform cell suspensions were obtained. The suspended cells were subsequently passed through a cell strainer and centrifuged at 400 × *g* for 10 min. Red blood cells were removed as described above. After being washed twice with 1× PBS (Invitrogen), the cell pellets were resuspended in sorting buffer (PBS supplemented with 1% FBS).

### Single-cell RNA-seq library and TCR-seq library preparation and sequencing

Single-cell RNA-seq libraries were prepared using the Chromium Next GEM Single-Cell 5′ Kit from 10× Genomics following the manufacturer’s instructions. In brief, single cells were washed once with PBS containing 2% fetal bovine serum (FBS) and resuspended in PBS containing 2% FBS to a final concentration of 500∼1200 cells/μl, as determined by Rigel S2 cell counter (Countstar). Cells (∼10,000) were captured in droplets to generate nanoliter-scale gel beads in emulsion (GEMs). The GEMs were then reverse transcribed in a C1000 Touch Thermal Cycler (Bio-Rad) programmed at 53 °C for 45 min and 85 °C for 5 min, followed by a hold at 4 °C. After reverse transcription and cell barcoding, the emulsions were broken, and cDNA was isolated and purified with Cleanup Mix containing DynaBeads and SPRIselect reagent (Beckman Coulter), followed by PCR amplification. The amplified cDNA was subsequently used for both 5′ gene expression library construction and TCR V(D)J targeted enrichment, which were performed with the Chromium Single-Cell V(D)J Enrichment Kit, Human T cell (10x Genomics), followed by V(D)J library construction. For RNA-seq library construction, amplified cDNA was fragmented and end-repaired, double-sided size selected, PCR-amplified with sample indexing primers, and double-sided size selected. For TCR-seq library construction, TCR transcripts were enriched from amplified cDNA by PCR. The enriched PCR product was subsequently fragmented and end-repaired, size-selected, PCR-amplified with sample-indexing primers, and size-selected. Libraries prepared in accordance with the manufacturer’s user instructions were then purified and profiled for quality assessment. Single-cell RNA and TCR V(D)J libraries were sequenced on an Illumina NovaSeq 6000 platform with 150 bp paired-end reads.

### Single-cell RNA-Seq data processing

The Cell Ranger toolkit (version 8.1.0) provided by 10× Genomics was used to aggregate the raw data, filter low-quality reads, align reads to the human reference genome (GRCh38), assign cell barcodes, and generate the unique molecular identifier (UMI) matrix.

An R-based toolkit, Seurat (version 5.3.0)^48^, was used to analyze the scRNA-seq data. Specifically, the raw UMI count matrix was processed to filter out genes detected in fewer than 10 cells and cells with fewer than 200 genes. We further quantified the numbers of genes and UMIs for each cell and kept high-quality cells with thresholds of 600–120,000 UMIs, 400–8000 genes and less than 10% mitochondrial gene counts to ensure that most of the heterogeneous cell types were included for downstream analyzes. Genes expressed in fewer than 0.1% of cells were removed. The normalized expression matrix was calculated on the basis of the raw UMI counts after the total counts per cell (library size) were normalized and then scaled by 1e5 and logarithmically transformed.

### Dimensionality reduction and unsupervised clustering for scRNA-seq data

Dimensionality reduction and unsupervised clustering were performed in accordance with the standard workflow in Seurat^48^. Briefly, the top 2000 highly variable genes (HVGs) were identified using vst-based methods with the default parameters. Principal component analysis (PCA) of the variable gene matrix was performed to reduce noise, and the top 50 components were used for downstream analyzes. To correct the batch effects from different patients, we used Harmony (version 1.2.0)^49^. Nearest neighbor graphs were subsequently generated to detect communities and identify cell clusters via the FindNeighbors function. Notably, the same principal components were also used for nonlinear dimension reduction to generate uniform manifold approximation and projection (UMAP) plots for visualization.

After the initial round of unsupervised clustering of all cells using the FindClusters function with a resolution of 1, we annotated each cell cluster on the basis of canonical immune cell markers (Supplementary Fig. 3) and identified the major cell types, including tumor cells (epithelial cells), stromal cells (mural cells, fibroblasts, endo cells, and myoepithelial cells), and immune cells (T cells, myeloid cells, NK cells, plasma cells, B cells, and mast cells).

We then performed a second round of unsupervised clustering of immune cells individually, at a resolution of 1, to refine the subset clusters. This clustering procedure was the same as the first-round clustering procedure and was initiated after batch effect correction with Harmony; cell clusters were detected by the FindNeighbors and FindClusters functions, and dimensionality reduction was performed for visualization.

We then examined the expression of well-characterized marker genes specific to various immune cell types (Supplementary Fig. 7) and the markers reported in the IMC dataset^21^. Through these strategies, we annotated our cell clusters and confirmed that they included commonly reported cell types from scRNA-seq studies.

### IMC data analysis

The IMC data were derived from a previously published study^21^ and obtained from Zenodo (https://doi.org/10.5281/zenodo.7990870) after requesting access.

For cell annotation and interaction inference, we used the annotation and neighbor information reported by the authors in the original article. Notably, neighbors indicate direct spatial contact between cells. The cluster labels were obtained from the “Label” column in the provided “cells.csv” file. The major cluster type labels (“TME”, “DCIS”, “invasive”, and “normal”) were obtained from the “cellAnnotation” column in the provided “cells.csv” file. The neighbor information between different cells was obtained from the provided “neighbours.csv” file.

For tumor cell analysis, we treated all epithelial cells as tumor cells and included the “DCIS”, “invasive”, and “normal” values in the “cellAnnotation” column. For immune cell fraction analysis, we included all immune cells with value of “TME” in the “cellAnnotation” column as the denominator. For interaction analysis and interaction index calculation, on the basis of the supplied neighbor information, we calculated the interaction index of the number of interactions between the epithelial cell phenotype and TME cell phenotype (heterotypic interactions) normalized by the total number of cells present.

### TCR sequence analysis

For TCR sequence assembly, the Cell Ranger toolkit (version 8.1.0) provided by 10× Genomics was applied to align the TCR-seq reads to the human reference genome (GRCh38) and assemble the TCR sequences.

An R-based toolkit, scRepertoire (version 2.5.1)^50,51^, was used to analyze the scTCR-seq data with the default parameters, unless otherwise specified. Cells with identical TCR pairs were defined as clonal cells and considered to originate from the same ancestry. TCR repertoire diversity was quantified at the patient level using Shannon diversity, calculated by the clonalDiversity function in scRepertoire with n.boots=1000. Shannon diversity captures both richness and evenness of the TCR repertoire, with higher values indicating greater diversity.

The clonalScatter function was used for scatter plot visualization to directly compare the specific clones among different treatments and HER2 status groups. The clones were categorized by counts into singlets or expanded colonies, either exclusive or shared between the selected samples. To quantify clonal expansion between pre-treatment and on-treatment samples, we then quantified clonal dynamics using the distance of each clone from the diagonal (*y* = *x*) in the scatter plot. Specifically, for each clone, the signed and absolute distances from the diagonal were calculated, reflecting directional change (expansion or contraction) and magnitude of change, respectively. At the patient level, we summarized these values using the median absolute distance across all clones, providing a robust measure of overall clonal redistribution.

We further aggregated the scTCR-seq data and scRNA-seq data for T cells for integration analysis. T cell type labels annotated on the basis of the scRNA-seq data were also appended to the integrated data. In total, 86.93% (172,903/198,901) of the T cells were assigned TCR sequences. Next, we assessed the distribution of the clone size of each T cell type in the different groups.

### Pseudobulk-based differentially expressed gene (DEG) analysis

To identify DEGs in specific cell types between different HER2 status groups from the single-cell data, a pseudobulk approach that accounts for patient-level variation was used. Cell-level differential expression analysis was not used to avoid inflated statistical significance. Specifically, single-cell RNA raw gene expression counts were summarized using the AggregateExpression function from R Seurat package^48^, grouping cells by cell type and sample identity. This approach generated a pseudobulk count matrix. Differential expression analysis was performed using DESeqDataSetFromMatrix function in R DESeq2 package^52^, with the HER2-low group as the experimental group and the HER2-0 group as the control group. *P* value and the average log2 FC (fold change) were used for visualization. Given the hypothesis-driven evaluation of a predefined gene set (genes that are associated with immunogenicity and were included in the IMC dataset), *p*-value was not adjusted, and nominal *p*-values are reported.

From the microarray transcriptome data from the ISPY-2 dataset, DEGs were identified using the limma (version 3.58.1) package^53^ on the basis of normalized expression data yielded by gNormalizedSignal (log2), and the *ERBB2* expression level and dataset ID (for adjusting batch effects) were set as the covariates.

From the bulk RNA-seq data included in the training and testing datasets used for the *ERBB2* expression prediction model, DEGs were identified using the eBayes function in the limma (version 3.58.1) package^53^ on the basis of the normalized FPKM/TPM (log2) values, and the *ERBB2* expression level and dataset ID (for adjusting batch effects) were set as the covariates.

### Statistical analysis

The clinicopathological and epidemiological characteristics of the HER2-low and HER2-0 subgroups are presented as medians for continuous variables and as frequencies for categorical variables. *P* values were calculated using Fisher’s exact test for categorical variables and the Wilcoxon rank-sum test for continuous variables. The odds ratio (OR) was calculated to determine differences in the pCR rate between the HER2-low and HER2-0 groups, and differences were compared via Fisher’s exact test. Fisher’s exact (Pearson’s χ2) test was used to compare the pCR rate between the HER2-low subgroup and the HER2-0 subgroup. *ERBB2* expression was compared between the pCR subgroup and the RD subgroup via the Wilcoxon rank-sum test.

To address potential confounders caused by the imbalance in characteristics between the HER2-low and HER2-0 groups and simulate the conditions of a randomized controlled trial, we performed 1:1 PSM without replacement in both the immunotherapy and chemotherapy cohorts. The propensity score, defined as the probability of a patient being assigned to the HER2-low or HER2-0 group given their baseline covariates, was estimated using a multivariable logistic regression model. The model included the following prespecified covariates believed to be associated with both the exposure and the outcome: grade, Ki67 expression, PD-L1 expression, CPS, cT stage, cN stage, and age. Missing values for PD-L1 expression were imputed with the median. Sensitivity analyzes with different imputation strategies (mean or zero) and excluding patients with missing PD-L1 test were also performed. Matching was performed using nearest neighbor matching (1:1 ratio) with a caliper width of 0.2 of the standard deviation of the logit of the propensity score using the matchit function in R without replacement to minimize imbalance. Fisher’s exact (Pearson’s *χ*2) test was then used to compare the pCR rate of the HER2-low subgroup and the HER2-0 subgroup in the matched cohort.

A linear regression model was fitted to examine the relationships between the predicted *ERBB2* expression and cluster proportions or marker expression levels in the IMC dataset. Given that the data might not meet all assumptions of parametric tests (e.g., normality of residuals), Spearman’s rank-order correlation coefficients and *p* values were further employed as nonparametric measures to evaluate the monotonic relationship between *ERBB2* expression and cluster proportions or marker expression levels. For this method, a correlation coefficient (${r}_{s}$) that ranges from −1 to +1, where values closer to |1| indicate a stronger monotonic association and the sign indicates the direction of the relationship (positive or negative), was calculated.

Paired comparisons of pre- and on-treatment samples were performed using the Wilcoxon signed-rank test for patient-level Shannon diversity derived from TCR data. Comparisons of patient-level median absolute distance between groups were performed using the Wilcoxon rank-sum test.

All tests were two-sided. *P* values < 0.05 were considered to indicate statistical significance unless otherwise specified. All the statistical analyzes were performed using R statistical software version 4.3.

## Supplementary information

Supplementary Information

## Data Availability

The single cell RNA-seq data generated in this study have been deposited in the National Genomic Data Center database (https://ngdc.cncb.ac.cn/bioproject/) under accession number PRJCA066115. The SCAN-B and ABiM datasets are available on the Mendeley Data website (https://data.mendeley.com/datasets/yzxtxn4nmd/4)^45^. The datasets from the TCGA are available at the cBioPortal website (www.cbioportal.org/). The GSE194040, GSE173839, and GSE163882 datasets were obtained from the Gene Expression Omnibus (GEO) database (http://www.ncbi.nlm.nih.gov/geo/). FUSCC bulk RNA-seq and clinical data are available from the Fudan Data Portal (https://data.3steps.cn/cdataportal/)^25–27^. The IMC dataset from the NeoTRIP trial can be accessed at zenodo (https://doi.org/10.5281/zenodo.7990870) following the procedure described in the original article^21^.

## Code availability

All data analysis and processing were conducted using published software packages whose details have been previously described and referenced within the Methods. Essential codes for data processing and analysis are available on the Github website (https://github.com/Ge526874/HER2_low_immunotherapy).
