# Supplementary material for: Distinct immune microenvironment in HER2-low triple-negative breast cancer underlies inferior response to immunotherapy
Source: NPJ Precis Oncol. 2026 Jun 17;10:371. doi: 10.1038/s41698-026-01562-3 (PMC13639012; doi:10.1038/s41698-026-01562-3)
Supplement: Supplementary file 1 [file 41698_2026_1562_MOESM1_ESM.pdf]

Distinct Immune Microenvironment in HER2-Low Triple-Negative Breast Cancer Underlies Inferior Response to Immunotherapy

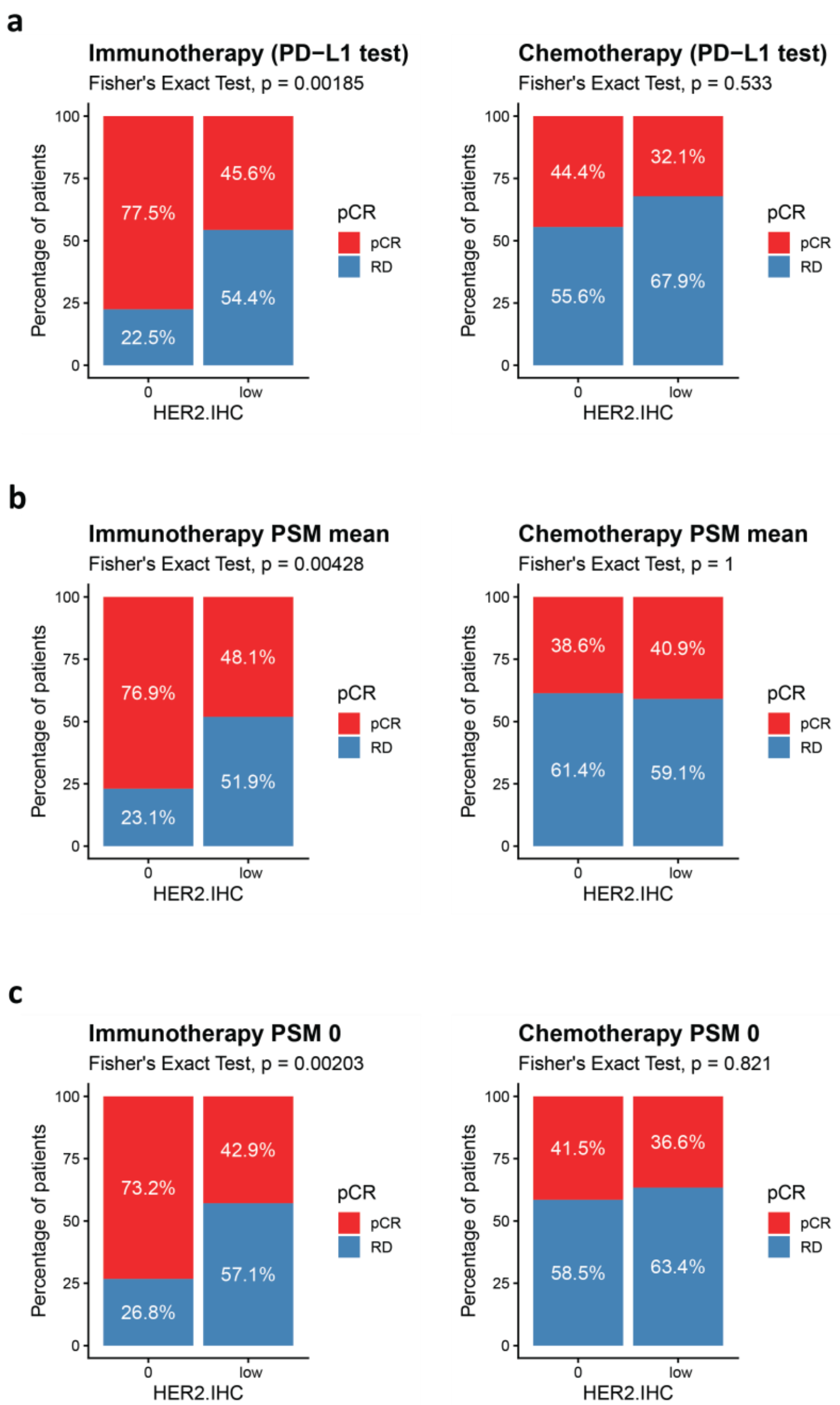

Supplementary Fig. 1 | **Effect of HER2 status on the response to neoadjuvant immunotherapy in institutional patients.** Response to immunotherapy combined with chemotherapy (left) and chemotherapy only (right) according to HER2 status after PSM. The missing PD-L1 test data were removed (**a**) or were imputed by mean (**b**) or 0 (**c**). Stacked bar chart showing the percentage of patients who achieved a pathological

complete response (pCR) versus those with residual disease (RD) after treatment, stratified by HER2 status. Statistical significance was assessed using Fisher's exact test.

pCR: pathological complete response

RD: residual disease

IHC: immunohistochemistry

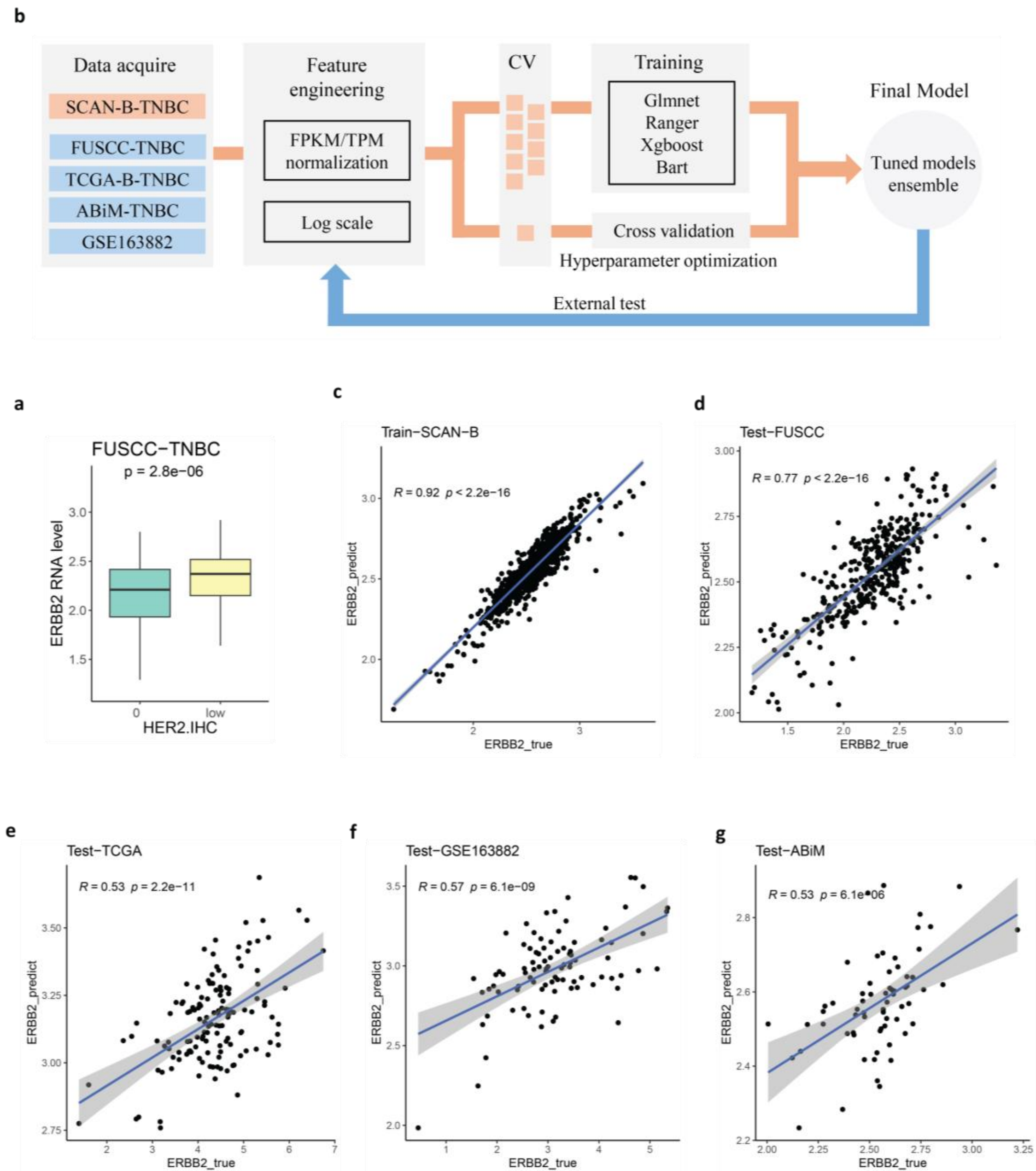

Supplementary Fig. 2 | **Machine learning model for predicting *ERBB2* RNA expression**. **a.** Comparison of *ERBB2* RNA expression by HER2 status in 359 TNBC patients using both RNA expression and HER2 IHC data from the FUSCC dataset. Box plots display the distribution of *ERBB2* expression in the HER2-0 and HER2-1+ groups. The centerline represents the median, the box limits represent the first and third quartiles (IQR), and the whiskers extend to the minimum and maximum values within 1.5 × IQR of the quartiles. The points beyond the whiskers are plotted individually as outliers. A

Wilcoxon rank-sum test was used to calculate the P value shown. **b.** Schematic of machine learning model construction and validation framework for *ERBB2* RNA expression level prediction. **d–g**. Correlations between predicted *ERBB2* expression and true *ERBB2* expression in the training SCAN-B dataset (**c**), FUSCC test set (**d**), TCGA test set (**e**), GSE163882 test set (**f**), and ABiM test set (**g**). Each point represents a sample in the scatter plot. A linear regression model (solid line) with a 95% confidence interval (shaded area) was fitted to the data. The strength and significance of the monotonic relationship were assessed using Pearson's correlation coefficient (R) and the P value.

CV: cross-validation

Glmnet: generalized linear model with elastic net regularization

Ranger: random forest

Xgboost: extreme gradient boosting

Bart: Bayesian additive regression tree

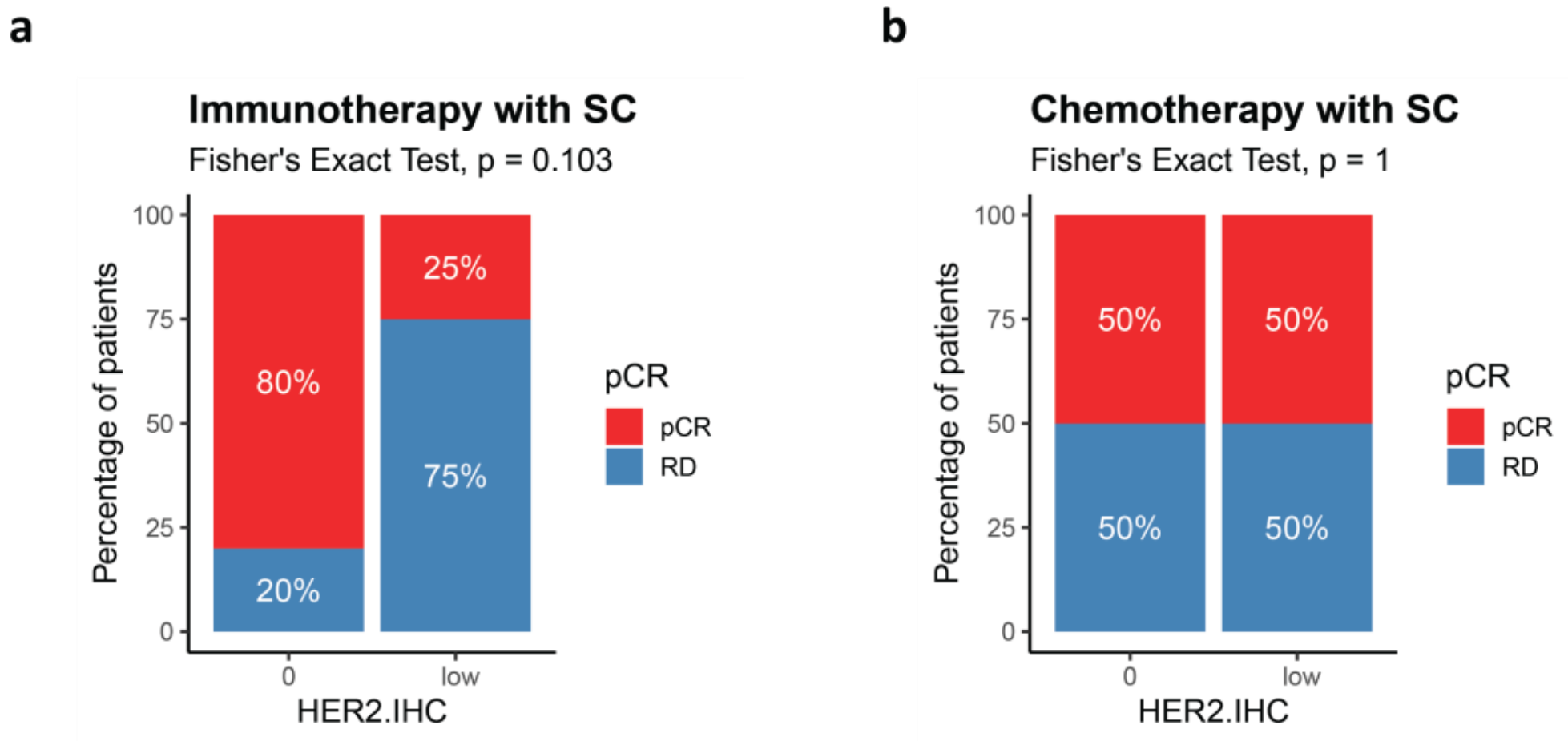

Supplementary Fig. 3 | **Effect of HER2 status on the response to neoadjuvant immunotherapy in patients with single-cell RNA-seq data. a-b.** Response to immunotherapy combined with chemotherapy (**a**) and chemotherapy alone (**b**) by HER2 status in patients with single-cell RNA-seq data.

pCR: pathological complete response

RD: residual disease

SC: single-cell

IHC: immunohistochemistry

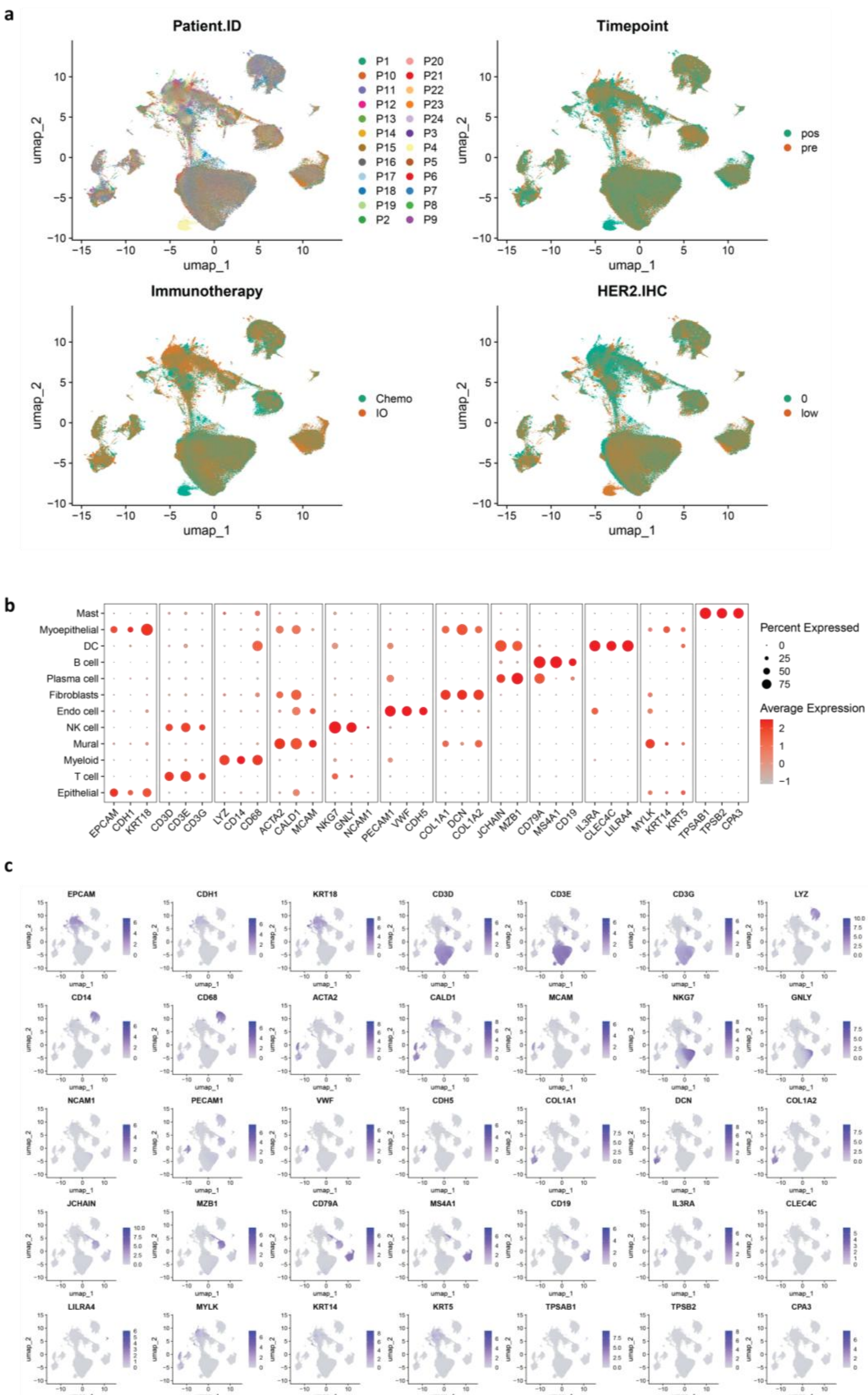
a
Patient.ID
Timepoint
Immunotherapy
HER2.IHC
umap_1
umap_2
P1 P10 P11 P12 P13 P14 P15 P16 P17 P18 P19 P2
P20 P21 P22 P23 P24 P3 P4 P5 P6 P7 P8 P9
pos
pre
Chemo
IO
0
low
b
Mast
Myoepithelial
DC
B cell
Plasma cell
Fibroblasts
Endo cell
NK cell
Mural
Myeloid
T cell
Epithelial
EPCAM CDH1 KRT18 CD3D CD3E CD3G LYZ CD14 CD68 ACTA2 CALD1 MCAM NKG7 GNLY NCAM1 PECAM1 VWF CDH5 COL1A1 DCN COL1A2 JCHAIN MZB1 CD79A MS4A1 CD19 IL3RA CLEC4C LILRA4 MYLK KRT14 KRT5 TPSAB1 TPSB2 CPA3
Percent Expressed
0
25
50
75
Average Expression
2
1
0
−1
c
EPCAM CDH1 KRT18 CD3D CD3E CD3G LYZ
CD14 CD68 ACTA2 CALD1 MCAM NKG7 GNLY
NCAM1 PECAM1 VWF CDH5 COL1A1 DCN COL1A2
JCHAIN MZB1 CD79A MS4A1 CD19 IL3RA CLEC4C
LILRA4 MYLK KRT14 KRT5 TPSAB1 TPSB2 CPA3

Supplementary Fig. 4 | **Characteristics of major cell types according to single cell data, Related to Figure 3. a.** UMAP plots showing the origins of individual cells within the TNBCs. Colors represent different sample origins. **b.** Bubble plots illustrating the expressions of selected marker genes in major cell subsets. **c.** UMAP plots showing the expressions of selected marker genes in major immune cell types.

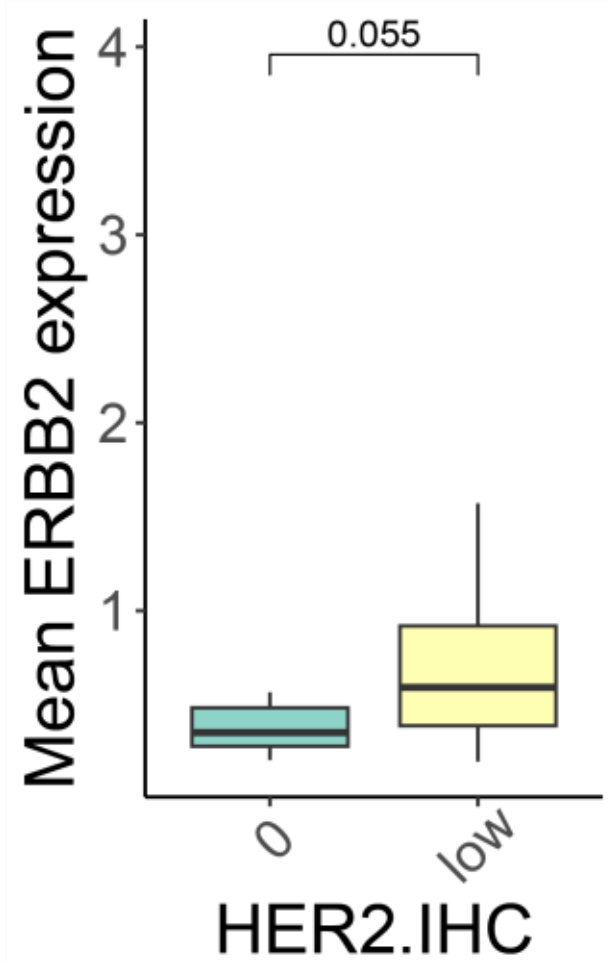

Supplementary Fig. 5 | **Box plot comparing mean *ERBB2* RNA expression in pretreatment tumor cells by HER2 status according to single-cell data.** Genes are labeled with functional type. The centerline represents the median, the box limits represent the first and third quartiles (IQR), and the whiskers extend to the minimum and maximum values within 1.5 × IQR of the quartiles. Points beyond the whiskers are plotted individually as outliers. A Wilcoxon rank-sum test was used to calculate the p-value shown.

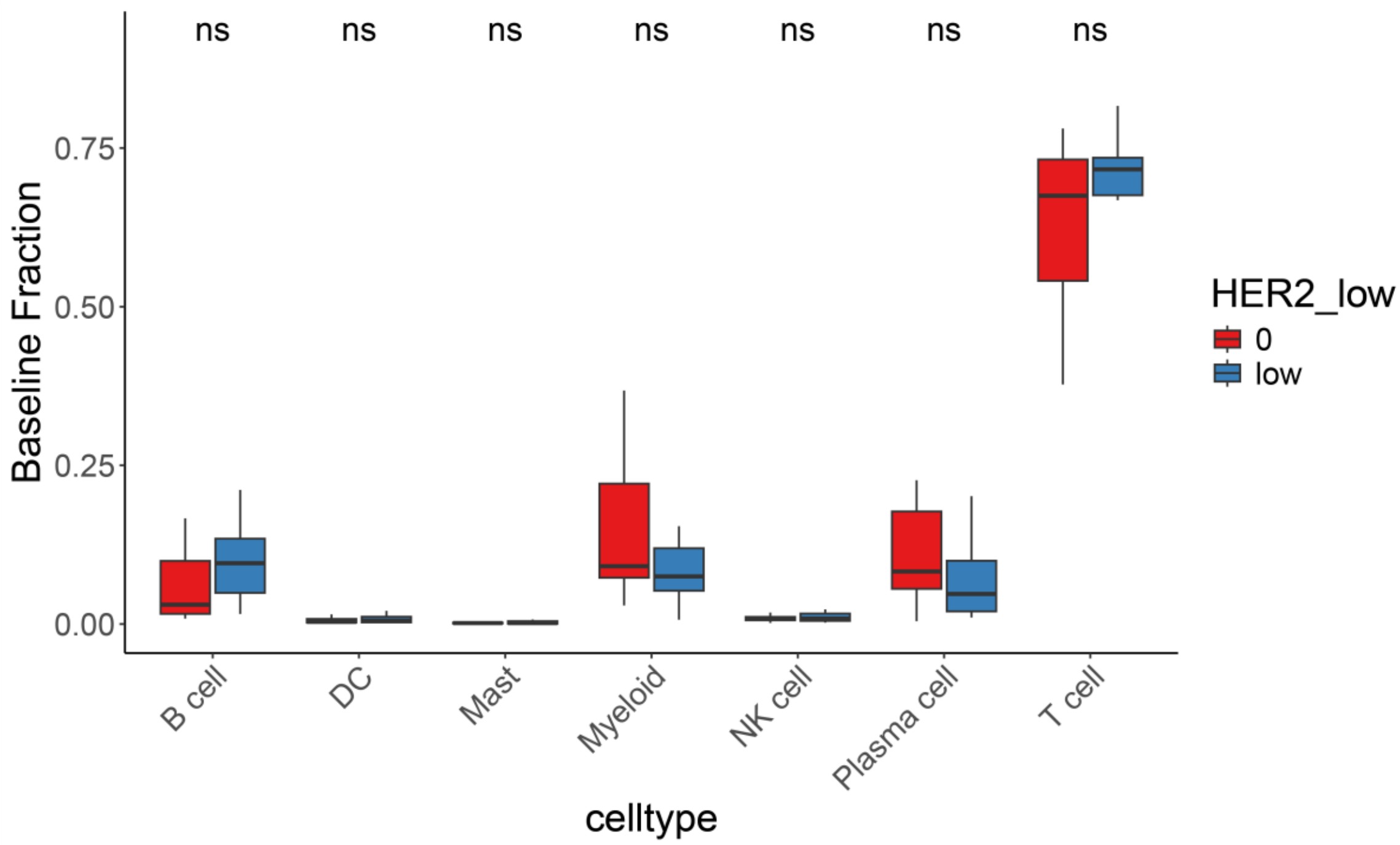

Supplementary Fig. 6 | **Baseline fraction of major immune cell types by HER2 status.** Box plots show the difference in major immune cell clusters proportions between the HER2-1+ and HER2-0 groups. The centerline represents the median, the box limits represent the first and third quartiles (IQR), and the whiskers extend to the minimum and maximum values within 1.5 × IQR of the quartiles. The Wilcoxon rank-sum test was used to calculate the P values shown. The significance levels are indicated above the plots (ns: not significant; *p < 0.05, **p < 0.01, ***p < 0.001, and ****p < 0.0001).

a

b

c

d

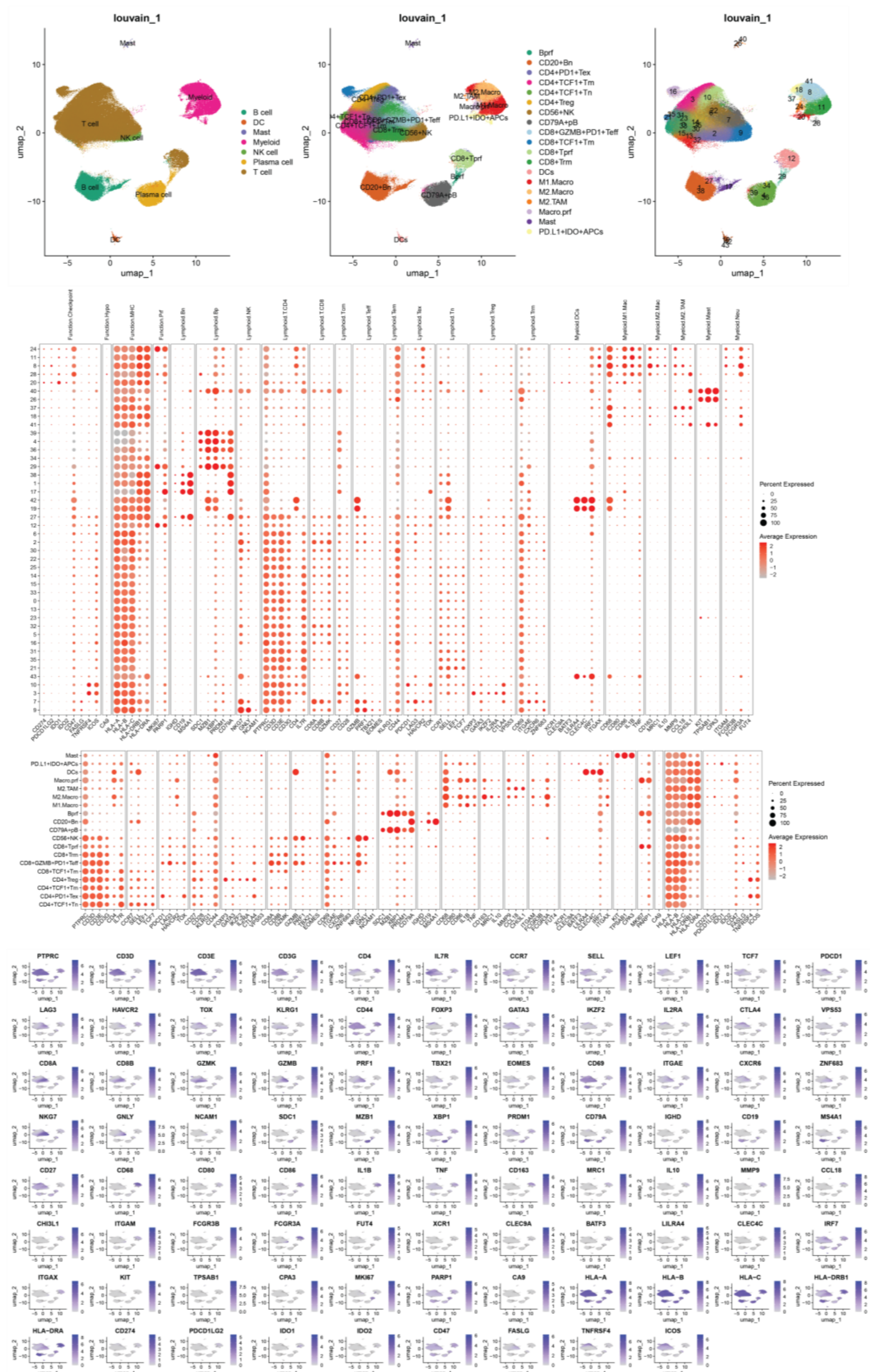
louvain_1
umap_1
umap_2
B cell
DC
Mast
Myeloid
NK cell
Plasma cell
T cell
Bprf
CD20+Bn
CD4+PD1+Tex
CD4+TCF1+Tm
CD4+TCF1+Tn
CD4+Treg
CD56+NK
CD79A+pB
CD8+GZMB+PD1+Teff
CD8+TCF1+Tm
CD8+Tprf
CD8+Trm
DCs
M1.Macro
M2.Macro
M2.TAM
Macro.prf
Mast
PD.L1+IDO+APCs
Percent Expressed
Average Expression
PTPRC
CD3D
CD3E
CD3G
CD4
IL7R
CCR7
SELL
LEF1
TCF7
PDCD1
LAG3
HAVCR2
TOX
KLRG1
CD44
FOXP3
GATA3
IKZF2
IL2RA
CTLA4
VPS53
CD8A
CD8B
GZMK
GZMB
PRF1
TBX21
EOMES
CD69
ITGAE
CXCR6
ZNF683
NKG7
GNLY
NCAM1
SDC1
MZB1
XBP1
PRDM1
CD79A
IGHD
CD19
MS4A1
CD27
CD68
CD80
CD86
IL1B
TNF
CD163
MRC1
IL10
MMP9
CCL18
CHI3L1
ITGAM
FCGR3B
FCGR3A
FUT4
XCR1
CLEC9A
BATF3
LILRA4
CLEC4C
IRF7
ITGAX
KIT
TPSAB1
CPA3
MKI67
PARP1
CA9
HLA−A
HLA−B
HLA−C
HLA−DRB1
HLA−DRA
CD274
PDCD1LG2
IDO1
IDO2
CD47
FASLG
TNFRSF4
ICOS

Supplementary Fig. 7 | **Characteristics of immune cell types in single cell data, Related to Figure 4. a.** UMAP plots showing immune cells for each cell type within the TNBCs. Colors represent different cell types. **b.** Bubble plots illustrating the expressions of selected marker genes in second round clustered immune cell subsets. **c.** Bubble plots illustrating the expressions of selected marker genes in second round annotated immune cell subsets. **d.** UMAP plots showing the expressions of selected marker genes (well-characterized marker genes and markers reported in the IMC dataset) in second round annotated immune cell types.

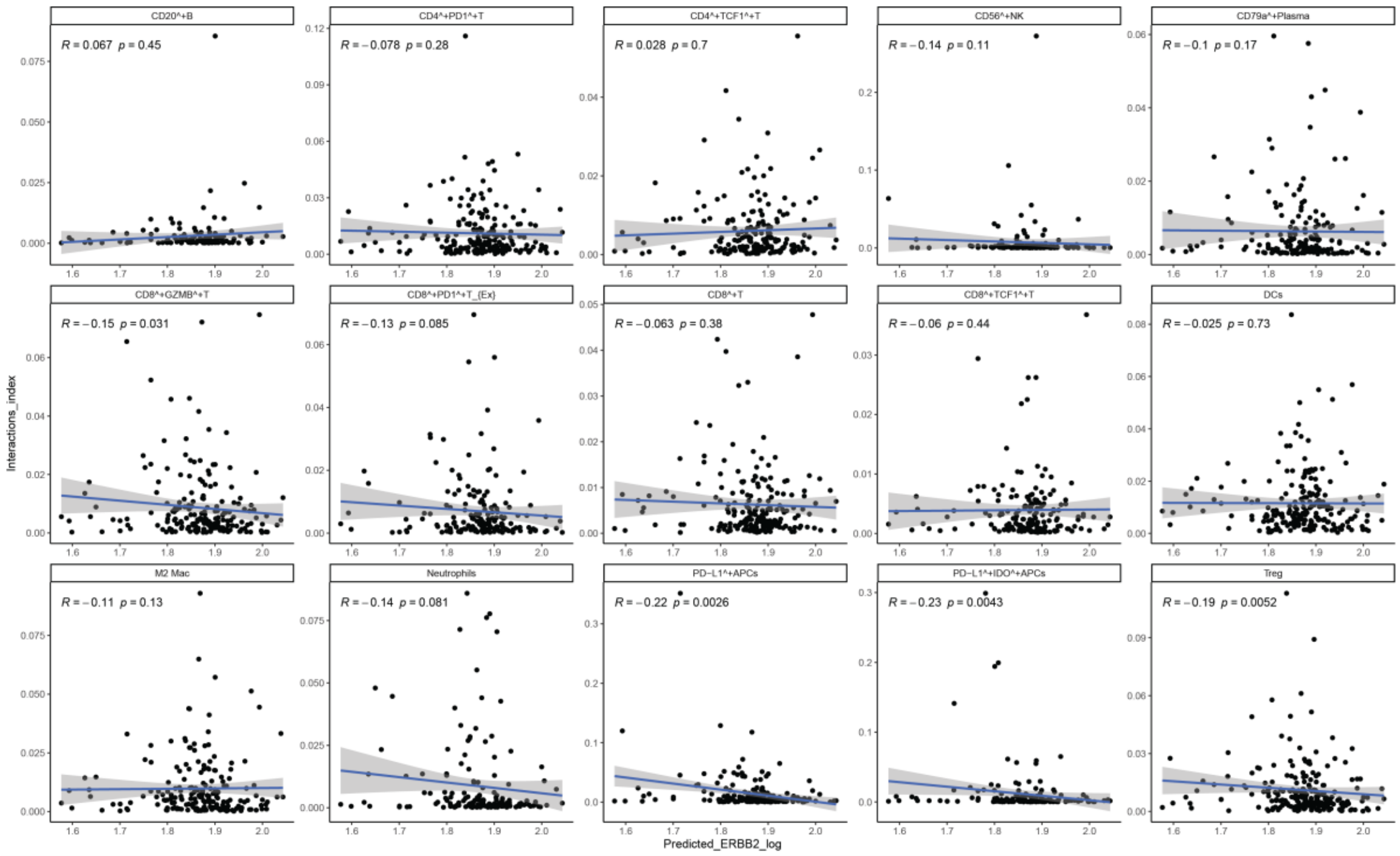

Supplementary Fig. 8 | **Correlation between predicted *ERBB2* expression and interaction indexes of immune cell types to epithelial cells according to the IMC dataset.** Each point represents a patient sample in the scatter plot. A linear regression model (solid line) with a 95% confidence interval (shaded area) was fitted to the data. The strength and significance of the monotonic relationship were assessed using Pearson's correlation coefficient (R) and the P value. The relationships between gene and protein markers (e.g., *MKI67* genes and Ki-67 proteins) are detailed in Supplementary Table 2.

a

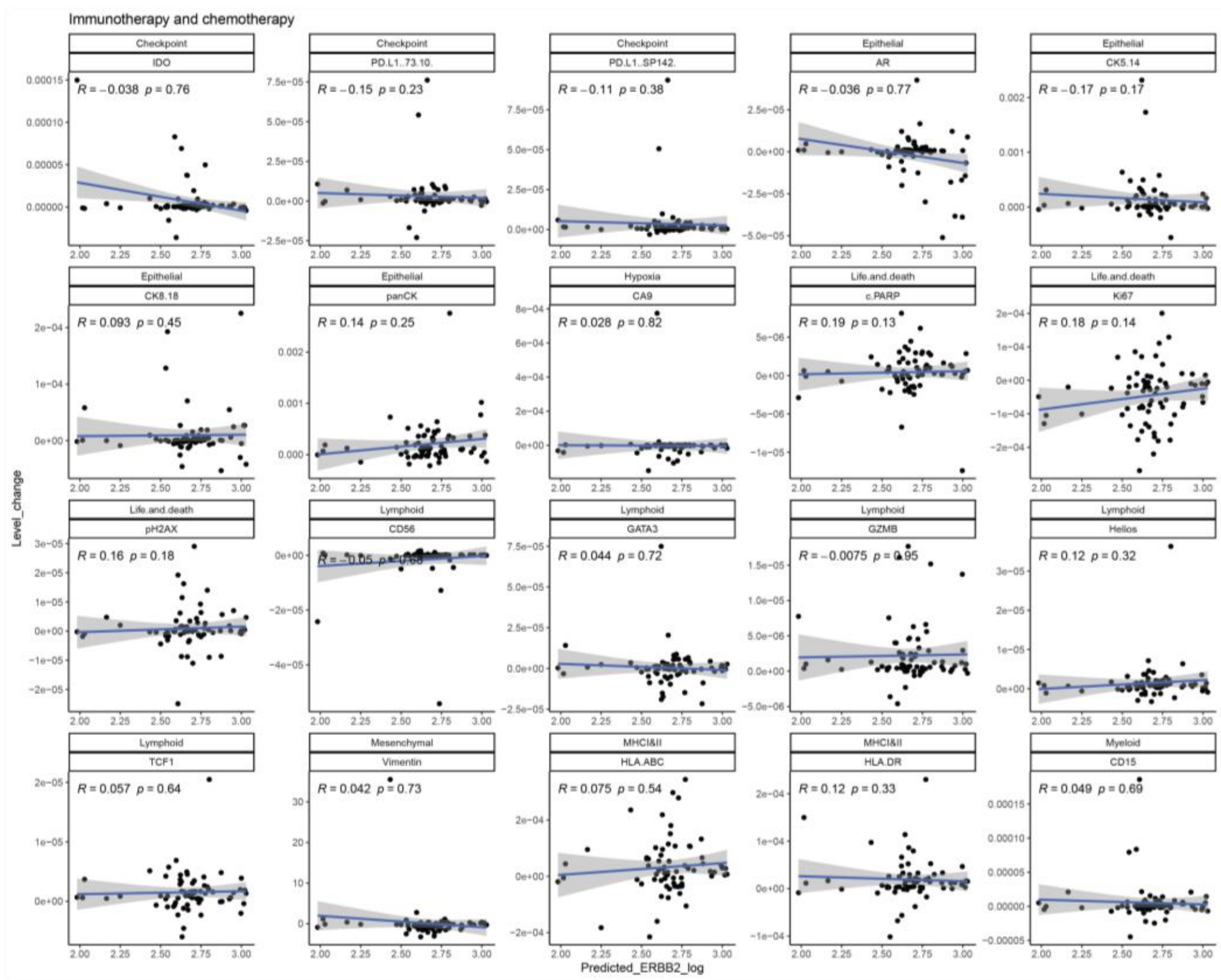
Immunotherapy and chemotherapy
Checkpoint IDO R = −0.038 p = 0.76
Checkpoint PD.L1..73.10. R = −0.15 p = 0.23
Checkpoint PD.L1..SP142. R = −0.11 p = 0.38
Epithelial AR R = −0.036 p = 0.77
Epithelial CK5.14 R = −0.17 p = 0.17
Epithelial CK8.18 R = 0.093 p = 0.45
Epithelial panCK R = 0.14 p = 0.25
Hypoxia CA9 R = 0.028 p = 0.82
Life.and.death c.PARP R = 0.19 p = 0.13
Life.and.death Ki67 R = 0.18 p = 0.14
Life.and.death pH2AX R = 0.16 p = 0.18
Lymphoid CD56
Lymphoid GATA3 R = 0.044 p = 0.72
Lymphoid GZMB R = −0.0075 p = 0.95
Lymphoid Helios R = 0.12 p = 0.32
Lymphoid TCF1 R = 0.057 p = 0.64
Mesenchymal Vimentin R = 0.042 p = 0.73
MHCI&II HLA.ABC R = 0.075 p = 0.54
MHCI&II HLA.DR R = 0.12 p = 0.33
Myeloid CD15 R = 0.049 p = 0.69
Level_change
Predicted_ERBB2_log

b

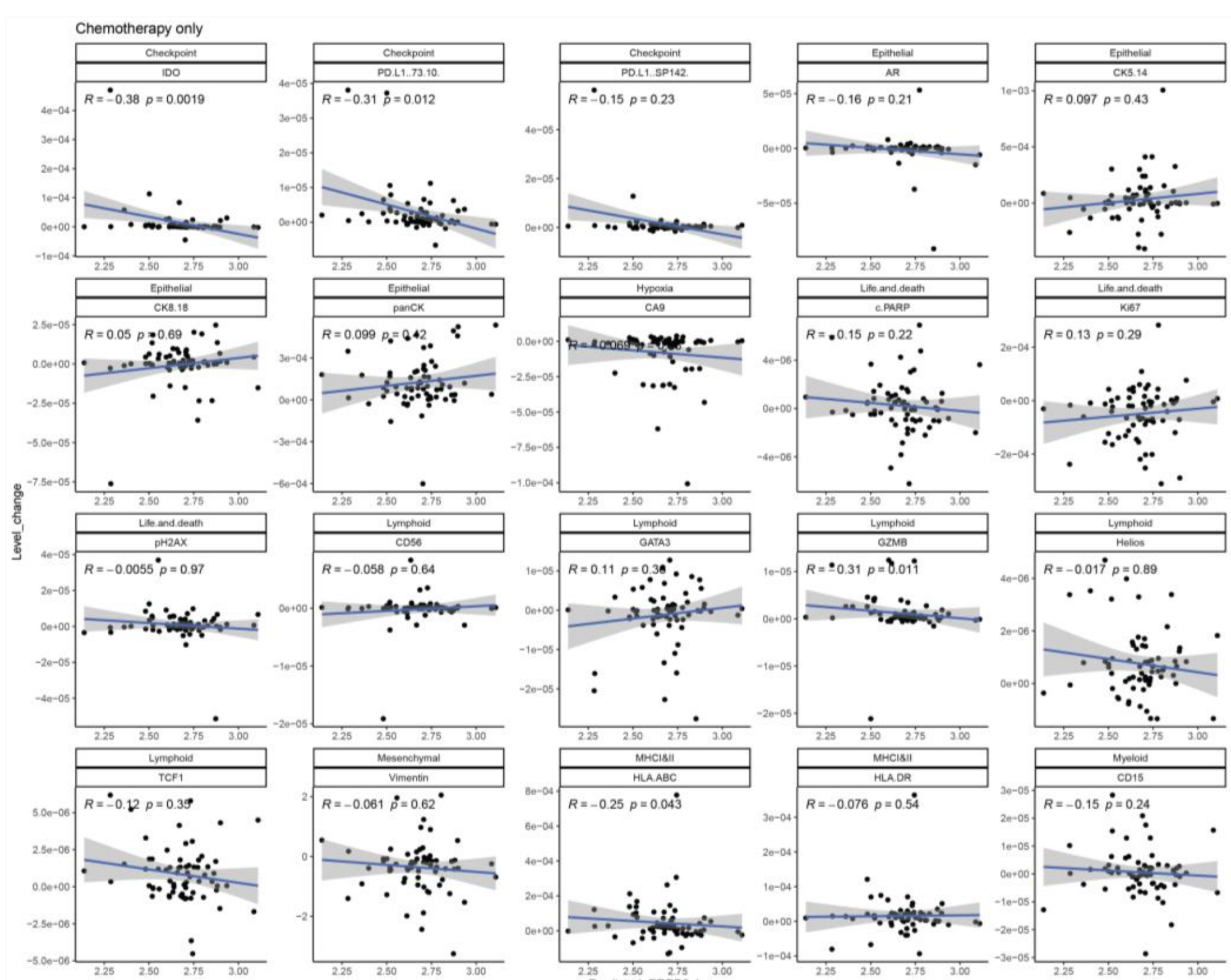
Chemotherapy only
Checkpoint IDO R = −0.38 p = 0.0019
Checkpoint PD.L1..73.10. R = −0.31 p = 0.012
Checkpoint PD.L1..SP142. R = −0.15 p = 0.23
Epithelial AR R = −0.16 p = 0.21
Epithelial CK5.14 R = 0.097 p = 0.43
Epithelial CK8.18 R = 0.05 p = 0.69
Epithelial panCK R = 0.099 p = 0.42
Hypoxia CA9
Life.and.death c.PARP R = −0.15 p = 0.22
Life.and.death Ki67 R = 0.13 p = 0.29
Life.and.death pH2AX R = −0.0055 p = 0.97
Lymphoid CD56 R = −0.058 p = 0.64
Lymphoid GATA3 R = 0.11 p = 0.38
Lymphoid GZMB R = −0.31 p = 0.011
Lymphoid Helios R = −0.017 p = 0.89
Lymphoid TCF1 R = −0.12 p = 0.35
Mesenchymal Vimentin R = −0.061 p = 0.62
MHCI&II HLA.ABC R = −0.25 p = 0.043
MHCI&II HLA.DR R = −0.076 p = 0.54
Myeloid CD15 R = −0.15 p = 0.24
Level_change
Predicted_ERBB2_log

Supplementary Fig. 9 | **Dynamic changes in protein markers in tumor cells according to the IMC dataset**. **a-b.** Correlation between predicted *ERBB2* expression and the mean changes in protein expression level of epithelial cells following immunotherapy combined with chemotherapy (**a**) and chemotherapy alone group (**b**). Proteins are labeled with functional type. Each point represents a patient sample in the scatter plot. A linear regression model (solid line) with a 95% confidence interval (shaded area) was fitted to the data. The strength and significance of the monotonic relationship were assessed using Pearson's correlation coefficient (R) and the P value. The relationships between gene and protein markers (e.g., *MKI67* genes and Ki-67 proteins) are detailed in Supplementary Table 2.

MHC: major histocompatibility complex

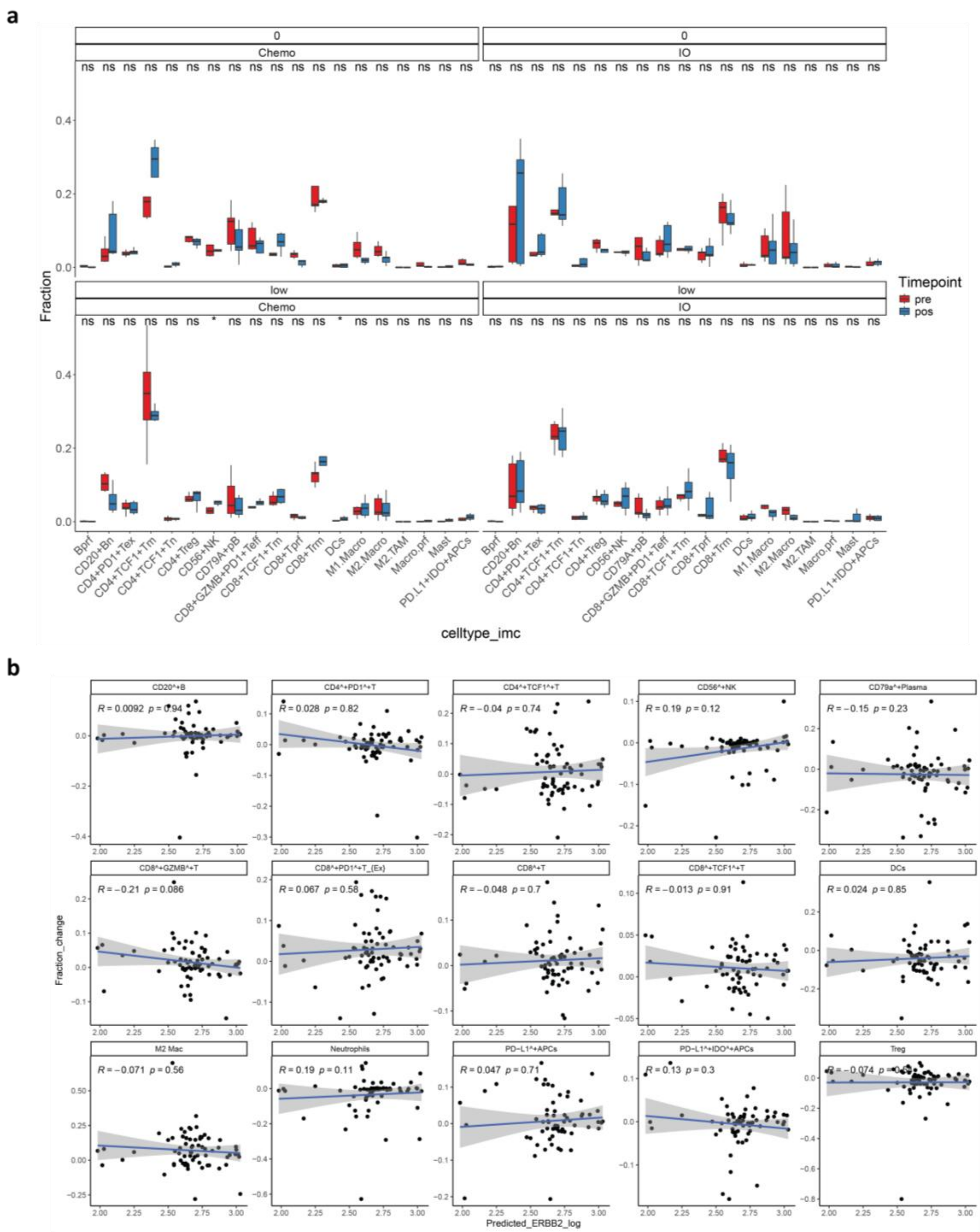

Supplementary Fig. 10 | **Dynamic changes in immune cells following immunotherapy. a.** Boxplots showing the difference of immune cell cluster proportions between the pre-treatment single-cell data and the on-treatment single cell data. The center line represents the median, the box limits represent the first and third quartiles (IQR), and the whiskers extend to the minimum and maximum values within 1.5 × IQR of the quartiles. Wilcoxon

rank-sum test was used to calculate the p-value shown. The significance levels are indicated above the plots (ns: not significant, *p < 0.05, **p < 0.01, ***p < 0.001, ****p < 0.0001). **b.** Correlation between patient predicted *ERBB2* expression and mean fraction change of immune cell clusters following immunotherapy and chemotherapy combination. Each point represents a patient sample in the scatter plot. A linear regression model (solid line) with a 95% confidence interval (shaded area) was fitted to the data. The strength and significance of the monotonic relationship were assessed using Pearson's correlation coefficient (R) and p-value.

The relationship between gene and protein markers (e.g., *MKI67* gene and Ki-67 protein) is detailed in Supplementary Table 2.

Chemo: Chemotherapy

IO: Immunotherapy

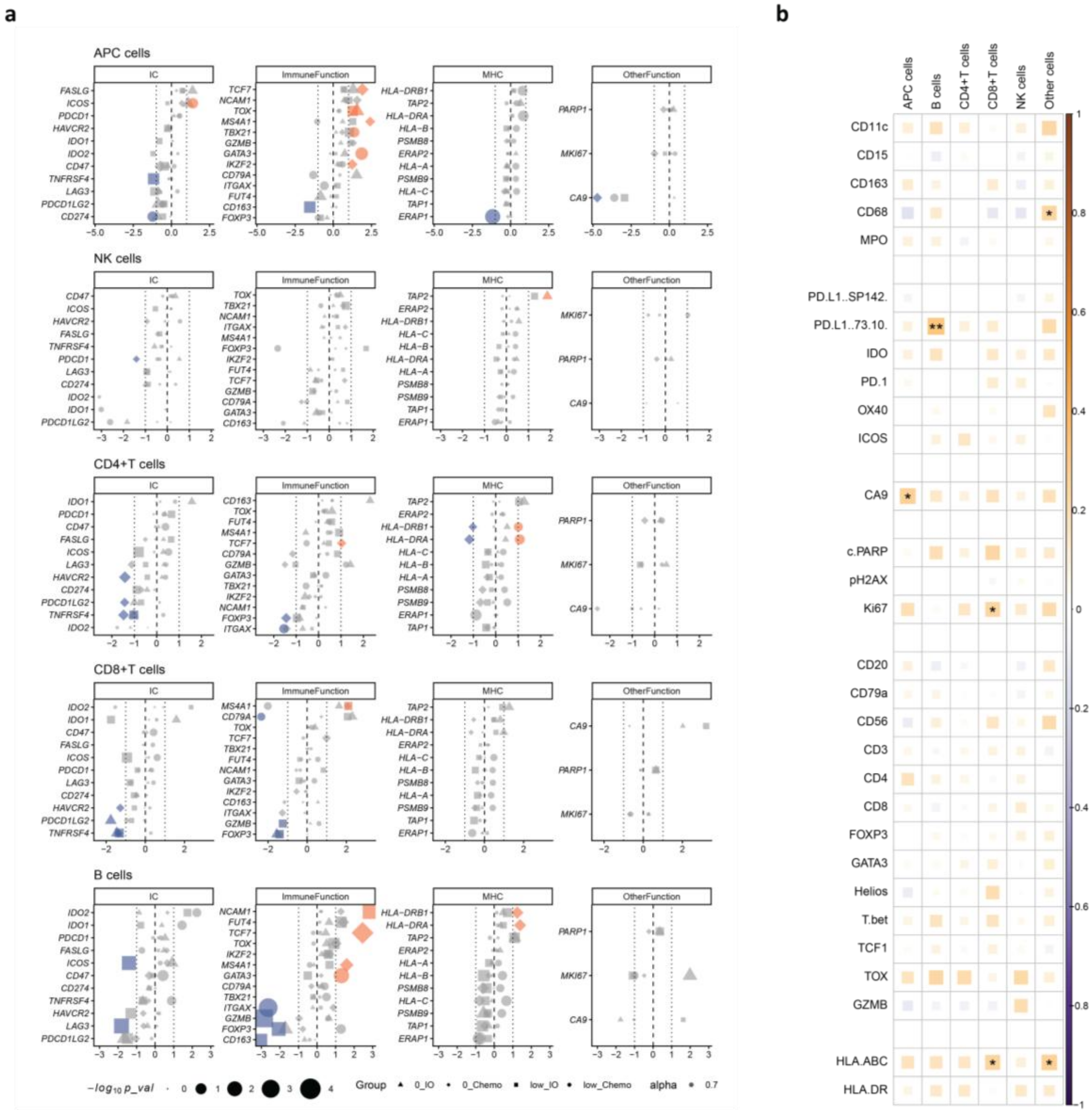

Supplementary Fig. 11 | **Dynamic changes in gene/protein markers in immune cells following immunotherapy. a.** Dot plot showing the pseudobulk-based differentially expressed genes in immune cells between baseline and on-treatment samples in the single cell dataset. Immune cells are grouped by cell type, and genes are grouped by functional type. Groups are labeled with various shapes. The x-axis represents the average log2-fold change in expression; positive values indicate higher expression in HER2-1+ tumor cells. The size of each point corresponds to its statistical significance (-log10(p_val)). Genes are colored on the basis of their fold change in expresion: markedly overexpressed (avg_log2FC ⩾ 1, orange), markedly underexpressed (avg_log2FC ⩽ -1, blue), or non-

marked (gray). The dashed and dotted lines indicate the zero-fold change and marked threshold, respectively. **b.** Correlation matrix between the change in the mean protein expression level in each immune cell cluster and the predicted *ERBB2* level. Proteins are separated by functional category. The color and size of each square represent the strength and direction of the Spearman correlation coefficient, with blue indicating a negative correlation and orange indicating a positive correlation. The statistical significance of each correlation is denoted by asterisks: ***$p < 0.001$, **$p < 0.01$, and *$p < 0.05$. Nonsignificant correlations ($p \geq 0.05$) are not marked. The relationships between gene and protein markers (e.g., *MKI67* genes and Ki-67 proteins) are detailed in Supplementary Table 2.

IC: immune checkpoint

MHC: major histocompatibility complex

Supplementary Table 1 | Baseline characteristics of patients in our retrospective study grouped by treatment types.

	Immunotherapy & chemotherapy			Chemotherapy alone		
	**HER2 0**	**HER2 1+**	**P**	**HER2 0**	**HER2 1+**	**P**
Number of patients	59	83		50	62	
Age (median (IQR))	46.00 [35.50, 55.00]	45.00 [36.25, 54.00]	0.945	44.50 [38.25, 54.00]	45.50 [37.25, 57.00]	0.699
cN (%)			0.098			0.668
cN0	16 (27.6)	19 (22.9)		11 (22.9)	12 (20.0)	
cN1	11 (19.0)	16 (19.3)		16 (33.3)	16 (26.7)	
cN2	19 (32.8)	16 (19.3)		14 (29.2)	18 (30.0)	
cN3	12 (20.7)	32 (38.6)		7 (14.6)	14 (23.3)	
cT (%)			0.215			0.265
cT0	1 (1.7)	2 (2.4)		0 (0.0)	2 (3.3)	
cT1	3 (5.1)	8 (9.8)		8 (16.7)	5 (8.2)	
cT2	35 (59.3)	53 (64.6)		22 (45.8)	30 (49.2)	
cT3	14 (23.7)	8 (9.8)		17 (35.4)	19 (31.1)	
cT4	6 (10.2)	11 (13.4)		1 (2.1)	5 (8.2)	
PD.L1.test	40 (67.8)	57 (68.7)	1	18 (36.0)	28 (45.2)	0.432
PD.L1.CPS (median (IQR))	20.00 [5.00, 42.50]	20.00 [5.00, 40.00]	0.906	7.50 [0.25, 27.50]	3.50 [0.00, 16.25]	0.615
Ki67 (median (IQR))	70.00 [50.00, 80.00]	70.00 [40.00, 80.00]	0.406	70.00 [60.00, 80.00]	65.00 [50.00, 80.00]	0.378
Grade (%)			0.584			0.733
Grade II	11 (21.2)	19 (27.1)		9 (22.5)	15 (27.8)	
Grade III	41 (78.8)	51 (72.9)		31 (77.5)	39 (72.2)	
Immunotherapy type (%)			0.554			
Anti-PD-1	53 (89.8)	78 (94.0)				
Anti-PD-L1	6 (10.2)	5 (6.0)				

Supplementary Table 2. The relationship between gene markers in the single cell dataset and protein markers in the IMC dataset.

**Type**	**Proteins**	**Genes**
Myeloid	CD11c	*ITGAX*
	CD15	*FUT4*
	CD163	*CD163*
	CD68	*CD68*
	MPO	*MPO*
Mesenchymal	Caveolin-1	*CAV1*
	CD31	*PECAM1*
	PDPN	*PDPN*
	PDGFRB	*PDGFRB*
	SMA	*ACTA2*

	Calponin	*CNN1*
	Vimentin	*VIM*
Checkpoint	PD-L1 (SP142)	*CD274*
	PD-L1 (73-10)	*CD274*
	IDO	*IDO1, IDO2*
	PD-1	*PDCD1*
	OX40	*CD134*
	ICOS	*ICOS*
Epithelial	AR	*AR*
Hypoxia	CA9	*CA9*
Life and death	c-PARP	*PARP1*
	Ki67	*MKI67*
Lymphoid	CD20	*MS4A1*
	CD79a	*CD79A*
	CD56	*NCAM1*
	CD3	*CD3D, CD3E*
	CD4	*CD4*
	CD8	*CD8A, CD8B*
	FOXP3	*FOXP3*
	GATA3	*GATA3*
	Helios	*IKZF2*
	T-bet	*TBX21*
	TCF1	*TCF7*
	TOX	*TOX*
	GZMB	*GZMB*
Pan-immune	CD45	*PTPRC*
MHC	HLA-ABC	*HLA-A, HLA-B, HLA-C*
	HLA-DR	*HLA-DRB1, HLA-DRA*
